# Palovarotene impact on fibrodysplasia ossificans progressiva (FOP): data from month 48 of the phase III MOVE trial

**DOI:** 10.1093/jbmrpl/ziag141

**Published:** 2026-08-29

**Authors:** Robert J Pignolo, Edward C Hsiao, Mona Al Mukaddam, Geneviève Baujat, Staffan K Berglund, Angela M Cheung, Carmen De Cunto, Patricia Delai, Nobuhiko Haga, Peter Kannu, Edna E Mancilla, Rose Marino, Fei Shih, Andrew Strahs, Frederick S Kaplan

**Affiliations:** Department of Medicine, Mayo Clinic, Rochester, MN 55905, United States; Division of Endocrinology and Metabolism, the UCSF Metabolic Bone Clinic, the Eli and Edyth Broad Institute for Regeneration Medicine, and the Institute of Human Genetics, Department of Medicine, and the UCSF Program in Craniofacial Biology, University of California San Francisco, San Francisco, CA 94143, United States; Departments of Orthopaedic Surgery and Medicine, Center for Research in FOP and Related Disorders, Perelman School of Medicine, University of Pennsylvania, Philadelphia, PA 19104, United States; Service de Médecine Génomique des Maladies Rares, Hôpital Necker-Enfants Malades, Paris 71015, France; Department of Clinical Sciences, Pediatrics, Umeå University, 901 87 Umeå, Sweden; Department of Medicine and Joint Department of Medical Imaging, University Health Network, University of Toronto, Toronto, ON M5G, Canada; Pediatric Rheumatology Section, Department of Pediatrics, Hospital Italiano de Buenos Aires, Buenos Aires C1199ACH, Argentina; Centro de Pesquisa Clinica, Hospital Israelita Albert Einstein, São Paulo 05652-000, Brazil; Department of Rehabilitation Medicine, The University of Tokyo Hospital, Tokyo 113-8655, Japan; Hospital for Sick Children, Toronto, ON M5G 1E8, Canada; Children’s Hospital of Philadelphia, Perelman School of Medicine, University of Pennsylvania, Philadelphia, PA 19104, United States; Ipsen, Cambridge, MA 02142, United States; Ipsen, Cambridge, MA 02142, United States; Ipsen, Cambridge, MA 02142, United States; Departments of Orthopaedic Surgery and Medicine, Center for Research in FOP and Related Disorders, Perelman School of Medicine, University of Pennsylvania, Philadelphia, PA 19104, United States

**Keywords:** fibrodysplasia ossificans progressiva, clinical trial, therapeutics, analysis/quantification of bone, growth plate

## Abstract

Palovarotene is approved in several countries for reducing heterotopic ossification (HO) in patients with fibrodysplasia ossificans progressiva (FOP), an ultra-rare, severely debilitating, genetic disorder. Approval was based on interim phase III MOVE trial (NCT03312634) analyses at month 18 (IA3), the most complete dataset considering subsequent clinical holds for futility and study restart; however, long-term data remain limited. We report exploratory, post hoc analyses of once daily palovarotene over the 48-mo MOVE trial, including time on and off treatment. The primary efficacy outcome was annualized change in new HO volume, per low-dose whole-body CT. Safety was assessed throughout. Post-baseline HO volume measurements were available for 97 palovarotene-treated patients from MOVE and 101 untreated patients from a natural history study (NHS; NCT02322255). Across 48 mo, mean annualized new HO volume was numerically lower (46.4%) in palovarotene-treated patients compared with untreated patients; however, this difference was not significant (nominal *p* = .0585). In patients who paused and stopped or paused and restarted palovarotene, mean annualized new HO volume appeared lower when patients received palovarotene compared with when they did not (significance not assessed). The proportions of patients with new HO and number of affected body regions were similar in palovarotene-treated patients and untreated patients (significance not assessed); flare-up rates were consistent with published literature. Adverse events were predominantly retinoid-associated mucocutaneous events. In 80 patients aged <18 yr in MOVE, palovarotene treatment occurred for a mean (SD) of 166.0 (75.2) wk; 24 (30.0%), all aged <14 yr, had premature physeal closure events. Computational analyses showed a significantly increased risk of radiographically-observed, asymptomatic new-onset vertebral deformities with palovarotene treatment. Overall, these exploratory analyses reaffirm the MOVE IA3 findings but should be interpreted with caution; potential efficacy must be weighed against the safety profile of palovarotene, particularly in growing patients. Clinical trial registration number: NCT03312634 (MOVE), NCT02322255 (NHS), and NCT02279095 (phase II OLE).

## Introduction

Fibrodysplasia ossificans progressiva (FOP; OMIM #135100) is an ultra-rare genetic disorder with an estimated prevalence of 1.39 per million individuals,^1^^,^^2^ caused by pathogenic variants in the Activin A Receptor Type 1 (*ACVR1*/*Activin Receptor-Like Kinase 2* [*ALK2*]) gene, most commonly *ACVR1*^R206H^.^3^^,^^4^ The pathogenic variants: confer increased basal activity to the ACVR1/ALK2 receptor,^5^ increase the activity of the receptor in response to binding of BMPs, and alter the response of the receptor to Activin A.^4^ These mechanisms lead to activation of downstream pathways and dysregulated transcription of genes involved in bone formation, causing cumulative and irreversible heterotopic ossification (HO) in soft connective tissues.^4–7^ HO occurs both chronically and episodically, with accumulation more marked around flare-ups.^8^^,^^9^ The development of ribbons, sheets, and plates of heterotopic bone progressively restrict movement, leading to cumulative disability, reduced quality of life, and shortened life expectancy.^10–13^

The current standard of care (SoC) for FOP is limited to symptom management and flare-up prevention.^14^^,^^15^ Palovarotene is an orally bioavailable retinoic acid receptor gamma agonist, which downregulates BMP pathway signaling to limit HO.^14^ To date, palovarotene has been approved for the reduction of new HO in adult and pediatric patients with FOP aged ≥8 yr for females and ≥10 yr for males by the Australian Therapeutic Goods Administration (TGA), Health Canada, the Japanese Pharmaceuticals and Medical Devices Agency, the Russian Federal Service for Surveillance in Healthcare (Roszdravnadzor), and the U.S. Food and Drug Administration (FDA); however, the European Medicines Agency (EMA) did not approve the marketing authorization application for palovarotene.^16–21^

Palovarotene has been evaluated in the largest global clinical program in FOP to date, including^22^: a randomized, double-blind, placebo-controlled phase II trial (NCT02190747)^23^; a phase II open-label extension (OLE; NCT02279095); an open-label phase III trial (MOVE; NCT03312634)^24^; and a phase III rollover trial (PIVOINE; NCT05027802). A prospective, non-interventional natural history study (NHS; NCT02322255) provided insight into FOP disease characteristics and natural progression in patients who were untreated beyond SoC.^9^ During the prespecified analysis at month 18 of the phase III MOVE trial (interim analysis 3; IA3), which represents the most complete dataset from this trial, no statistically significant difference was found in the mean volume of annualized new HO in patients treated with palovarotene in MOVE compared with untreated patients from the NHS, leading to a temporary suspension of the study.^24^ However, following unblinding, there was concern regarding limitations of the prespecified analysis.^24^ Subsequent post hoc analyses showed that the mean annualized new HO volume was 54% lower in MOVE compared with the NHS (weighted linear mixed effects [wLME] analysis, *p* = .039), supporting the approval of palovarotene in patients with FOP in several countries.^24^ Adverse events (AEs) in MOVE were consistent with other systemic retinoids; however, there was an increased risk of premature physeal closure (PPC) in patients aged <14 yr and a potential risk of decreased BMD and vertebral deformities or fractures.^24^

Here, we report supportive post hoc exploratory analyses from the entire 48-mo period of the phase III MOVE trial, supplemented by additional data from the phase II OLE,^22^ thus representing the totality of available evidence assessing the efficacy and safety of long-term palovarotene treatment in patients with FOP.

## Materials and methods

### Patients and trial design

MOVE (NCT03312634; registered October 18, 2017) was a multicenter, single-arm, open-label, phase III trial of palovarotene in patients with FOP; full methodology and patient demographics have been reported previously.^24^ Briefly, MOVE was conducted in 3 parts: Part A, the main part of the trial up to 24 mo (including IA 1-3)^24^; Part B, a 24-mo extension; and Part C, a 2-yr follow-up in skeletally immature patients who discontinued treatment before completion of Part A or B. No dosing occurred in Part C, which included yearly visits up to 2 yr following the last dose of palovarotene; whole-body CT (WBCT) assessments continued at Year 1 and 2 post-treatment. The maximum duration of participation could not extend beyond 48 mo for Parts A, B, and C in total; participation in Part C continued only as long as patients remained skeletally immature.

Patients aged ≥4 yr with a clinical diagnosis of FOP were eligible for inclusion, including patients who participated in the FOP NHS (NCT02322255; registered December 23, 2014).^24^ Patients received 5 mg oral palovarotene once daily. Upon the onset of any eligible flare-up (defined previously),^24^ patients received palovarotene 20 mg once daily for 4 wk, followed by 10 mg once daily for 8 wk. At the discretion of the Investigator, flare-up treatment could be extended in 4-wk intervals until flare-up resolution.^24^

Palovarotene dosing was weight-adjusted in patients aged <18 yr with <90% skeletal maturity on hand/wrist radiograph at screening.^24^ Palovarotene dosing could be reduced in patients who experienced intolerable AEs that would otherwise result in discontinuation, or discontinued if patients were already receiving the lowest dose.^24^

**Figure 1:**
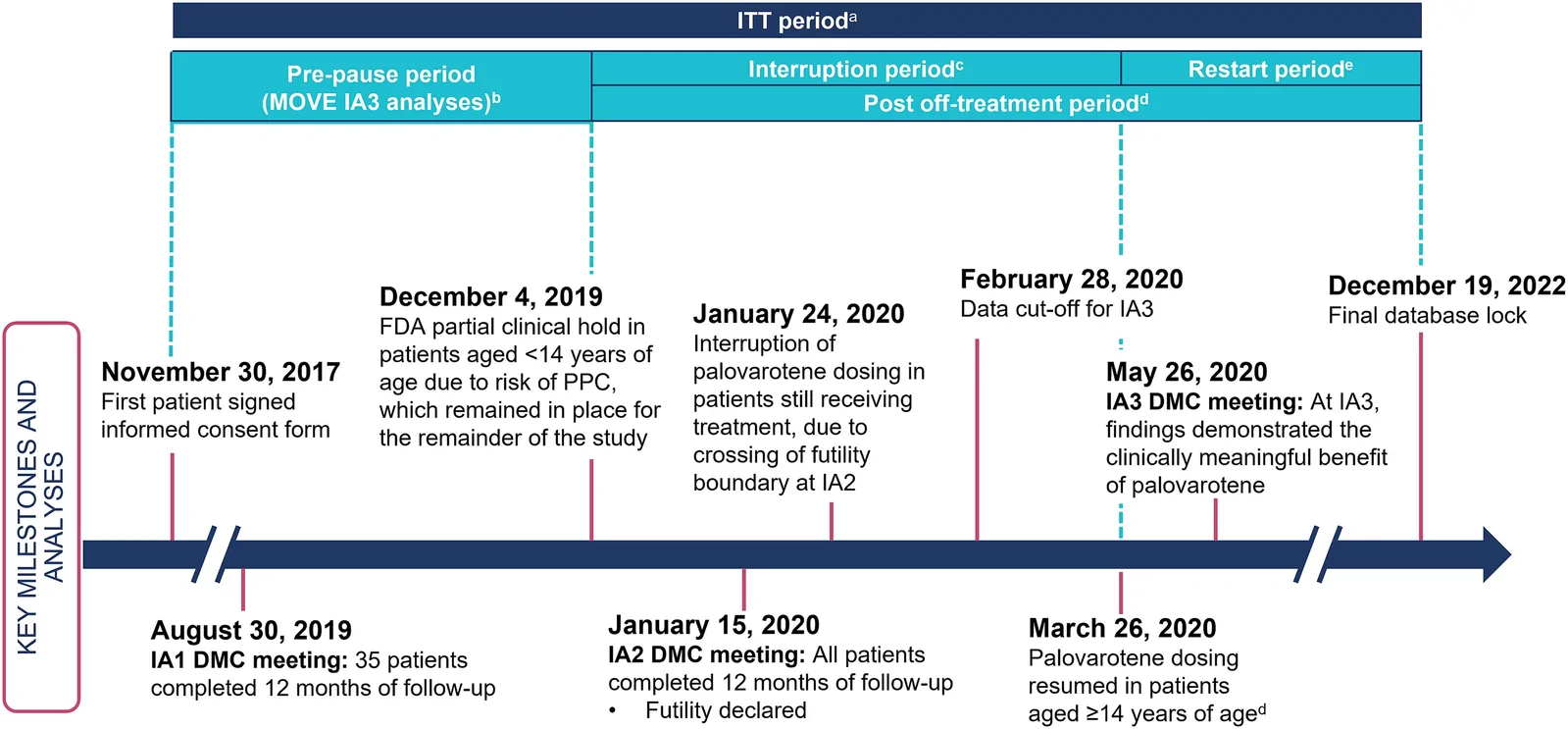
MOVE ITT analysis periods to trial completion. In the first 24 mo of the trial three interim analyses were planned: IA1 when 35 patients completed month 12, IA2 when all patients completed month 12, and IA3 when all patients completed month 18. Patient numbers varied across the study periods, due to the requirement for at least one baseline and one post-baseline HO volume measurement in each period. For the interruption period, restart period, and post off-treatment period, the WBCT scan for the baseline HO volume measurement could not have occurred during or within 4 wk of an active flare-up. ^a^Overall analyses encompassing time from screening through last WBCT scan; spans both on and off treatment, regardless of whether patients restarted or remained off palovarotene treatment; ^b^Spans the start of the study to dosing interruption (due to the partial clinical hold or the interruption due to passing the futility boundary). ^c^Spans the interruption due to the partial clinical hold and crossing the futility boundary, in patients who restarted palovarotene. ^d^Spans the period from first WBCT scan off-treatment following dosing interruption, in patients who remained off treatment. ^e^Spans the time spent on treatment following interruptions with ≥2 WBCT scans post-restart, in patients who restarted palovarotene. DMC, data monitoring committee; IA1, interim analysis 1; IA2, interim analysis 2; IA3, interim analysis 3; ITT, intent-to-treat; WBCT, whole-body CT.

In this analysis, data from the entire 48 mo of MOVE are presented from the intent-to-treat (ITT) period, which encompassed screening through to the last WBCT scan (final database lock: December 19, 2022) and included time on and off treatment (Figure 1). Two treatment interruptions occurred during MOVE.^24^ The first occurred following a partial clinical hold issued by the United States FDA in patients aged <14 yr due to the identified risk of PPC (December 4, 2019). This partial clinical hold remained in place for the remainder of the study; patients could not restart palovarotene treatment until they reached 14 yr of age. The second occurred in patients aged ≥14 yr following outcomes at IA2 that crossed the prespecified threshold for futility (January 24, 2020). As it was not possible to conclude treatment futility based on data at IA2, an independent data monitoring committee recommended the study continue on March 26, 2020, providing Ethics Committee approval for each site.^24^

Owing to these 2 interruptions, efficacy analyses were performed within distinct time periods (Figure 1). Analyses were conducted for: all patients in the ITT period, regardless of treatment status, compared with untreated patients from the NHS; palovarotene-treated patients who paused and then subsequently stopped treatment (post off-treatment period; defined as the period from first WBCT scan off treatment following dosing interruption to last patient, last visit for patients who remained off treatment); and palovarotene-treated patients who paused and restarted treatment, with data in each of the pre-pause, interruption, and restart periods (≥2 WBCT scans post-restart).

The trial design for the phase II OLE (NCT02279095; registered October 30, 2014) has been described previously^22^; a top-line overview is given in Supplementary Appendix 1.1.

### Efficacy outcomes in MOVE

The primary efficacy outcome of the MOVE trial was mean annualized change in new HO volume, assessed by low-dose WBCT (excluding the head). Assessment of changes in new HO volume encompassed formation from both new and existing HO lesions. Data were compared with those from the NHS, in which patients were untreated beyond SoC, and between the distinct time periods in MOVE described above. WBCT scans were obtained at baseline and months 6, 12, 18, and 24 in Part A of MOVE, at months 36 and 48 in Part B, and at the year 1 and year 2 follow-up visits in Part C.^24^ In the NHS, WBCT scans were obtained at baseline and every 12 mo.^9^ Interpretation of WBCT scans qualitatively documented the volume of total body HO, the presence or absence of HO across body regions, and the presence of new HO at follow-up visits. All images were interpreted by a central imaging core laboratory using standardized procedures.

Secondary and exploratory efficacy outcomes reported include: the proportion of patients who developed any new HO, the mean number of body regions with new HO, assessed by WBCT and based on 9 anatomical regions,^24^ and flare-up rate per patient-month of exposure, with flare-ups defined by ≥2 flare-up symptoms. Range of motion was assessed using the Cumulative Analogue Joint Involvement Scale (CAJIS) by trained Investigators; higher scores indicate worse joint mobility.^25^ Physical function was assessed using age-appropriate forms of the FOP-Physical Function Questionnaire (FOP-PFQ); results are expressed as a percentage of the worst possible score (0%-100%); higher scores indicate more severe limitations in physical functioning.^26^ The Patient-Reported Outcomes Measurement Information System (PROMIS) was used to assess physical and mental health impacts of FOP.^24^

### Safety outcomes in MOVE

AEs were monitored throughout MOVE and categorized as mild, moderate, or severe, according to the Medical Dictionary for Regulatory Activities (MedDRA; version 22.0). Serious AEs (SAEs) were those resulting in death or threat to life, hospitalization, significant disability, congenital birth defects, or any other medically important events.^24^ Additional safety monitoring has been described previously.^24^

### Incidence and management of skin AEs in MOVE

For post hoc analyses of the incidence, duration, intensity, and management of skin treatment-emergent AEs (TEAEs) in MOVE, mucocutaneous events were recorded based on MedDRA Preferred Term (version 22.0), characterized using the Standardized MedDRA Query of Severe Cutaneous Adverse Reactions (broad and narrow), and graded using the Common Terminology Criteria for Adverse Events (version 4.03). Patients in the MOVE Principal Safety population who had at least one skin TEAE during the trial were included in these post hoc analyses. Skin TEAEs were defined by a “skin and subcutaneous disorders” MedDRA System Organ Class or “lip dry” MedDRA Preferred Term. When mucocutaneous events were observed in MOVE, symptomatic therapies (eg, analgesics, skin emollients, lip moisturizers, artificial tears, or other helpful treatments) could be administered if deemed necessary by the Investigator. In addition, the Investigator could recommend prophylactic use of these therapies at the start of palovarotene treatment. Prophylactic medications that started or were ongoing 14 d prior to the start date of the first skin TEAE, and concomitant medications that started within 3 d of skin TEAE onset, are reported. The incidence, duration, intensity, and management of skin TEAEs with and without prophylactic treatment are also reported.

### P‌PC in MOVE and the phase II OLE

Careful assessments of epiphyseal abnormalities were included in the palovarotene clinical program. Physeal status was assessed by hand/wrist and knee radiographs every 6 mo in MOVE and the phase II OLE, in patients aged <18 yr with open epiphyseal plates. Radiograph frequency was increased to every 3 mo following observation of PPC events in patients with open epiphyseal plates who received flare-up treatment; once patients achieved 100% skeletal maturity, radiographs were no longer required. PPC events were recorded based on MedDRA (version 22.0) and included partial (growth plate has a definite disruption of portions of the adjacent outlines of the epiphyseal and metaphyseal regions) or complete (any portion of the growth plate with evidence of closure) growth plate closure. All PPC events were determined by the Investigator and categorized as SAEs, regardless of whether criteria for seriousness were met, and were evaluated from screening through January 2022.

Patients aged <18 yr with open epiphyseal plates also had linear growth assessments by stadiometer and knee height measurements every 6 mo; *z*-scores were calculated using U.S. Centers for Disease Control and Prevention growth charts.

### Biomechanical CT analyses in MOVE and the phase II OLE

Using finite element analysis, biomechanical CT (VirtuOst software)^27^ was performed retrospectively on WBCT scans to evaluate spinal (L1, L2, or adjacent vertebrae; 2 vertebral levels per patient) BMD, BMC, and bone strength annually from baseline in MOVE and the phase II OLE, compared with data from the NHS. Outcomes were assessed at baseline for palovarotene-treated and untreated patients, and across the duration of the palovarotene-exposed period and untreated periods in MOVE and the phase II OLE. For patients who crossed over from Part B to Part C in the phase II OLE, the baseline visit in Part B was considered baseline. Per Genant’s criteria,^28^ the degree of radiographically observed, asymptomatic vertebral deformities/fractures was assessed at all evaluable vertebrae between T4 through L4 and classified as: normal (Grade 0; deformity <20%), mild (Grade 1; deformity 20%-<25%), moderate (Grade 2; deformity 25%-<40%), or severe (Grade 3; deformity ≥40%).

### Statistical analyses

Sample size for the MOVE trial was determined as previously described.^24^ For efficacy analyses, the MOVE Principal Full Analysis Set (Principal FAS) included all enrolled patients with the *ACVR1*^R206H^ variant who had a baseline and at least one post-baseline HO volume measurement. For efficacy comparisons with the NHS, the Principal FAS also included patients enrolled in the NHS with available baseline and at least one post-baseline HO volume measurement.

The majority of efficacy and safety analyses are reported descriptively, and statistical significance was not assessed, due to their post hoc nature and the incomplete follow-up of patients in the trial. Where appropriate, two-sided 95% CIs and SDs are reported.

For comparison of the primary efficacy outcome during the MOVE ITT period with untreated patients from the NHS, a wLME model was applied, consistent with post hoc analyses conducted at MOVE IA3.^24^ Inferential analyses were performed, and a nominal *p*-value is reported for this comparison; however, due to the post hoc nature of these analyses, they must be interpreted with caution. To support findings at IA3,^24^ post hoc sensitivity analyses were performed for the wLME mean annualized new HO volume changes previously reported.^24^ The statistical analysis methods are described in Supplementary Appendix 1.2.

Adjusted regression models were used to assess the relationship between vertebral body strength, entire vertebral body BMC, and mid-vertebral (trabecular) BMD, and exposure to palovarotene in patients in MOVE and the phase II OLE vs untreated patients from the NHS. Similarly, adjusted Poisson regression models were used to assess the relationship between vertebral deformities/fractures and exposure to palovarotene. Characteristics adjusted for included baseline CAJIS score, age-adjusted baseline HO volume, sex, age at FOP onset, and steroid use.

## Results

### Patient disposition

Baseline demographics and disease characteristics of patients in MOVE and the NHS have been reported previously and were generally balanced,^24^ though a greater proportion of patients were aged <18 yr in MOVE (74.8%) compared with the NHS (57.9%). Patients included in the final analyses of MOVE had a similar general medical history and history of FOP and flare-ups, to those included in the MOVE IA3 analyses.^24^ In total, 39 patients transferred from the NHS to MOVE, and contributed data to both trial groups as post-baseline WBCT scans were available from both studies.

Overall, 97 patients from MOVE and 101 participants from the NHS who had a baseline and at least one post-baseline HO volume measurement were included in the principal efficacy analyses. In the MOVE ITT period, patients received palovarotene for a mean (SD) of 25.4 (12.5) mo and were off-treatment for 13.1 (9.3; range: 3.19–23.72) mo; the maximum dosing duration was approximately 44.9 mo and a total of 49 patients remained in the MOVE study for ≥48 mo.

### Primary efficacy outcome in MOVE

Post hoc sensitivity analyses of wLME mean annualized new HO volume changes at MOVE IA3 support the previously reported results (Table S1 and Figure S1).^24^

**Figure 2:**
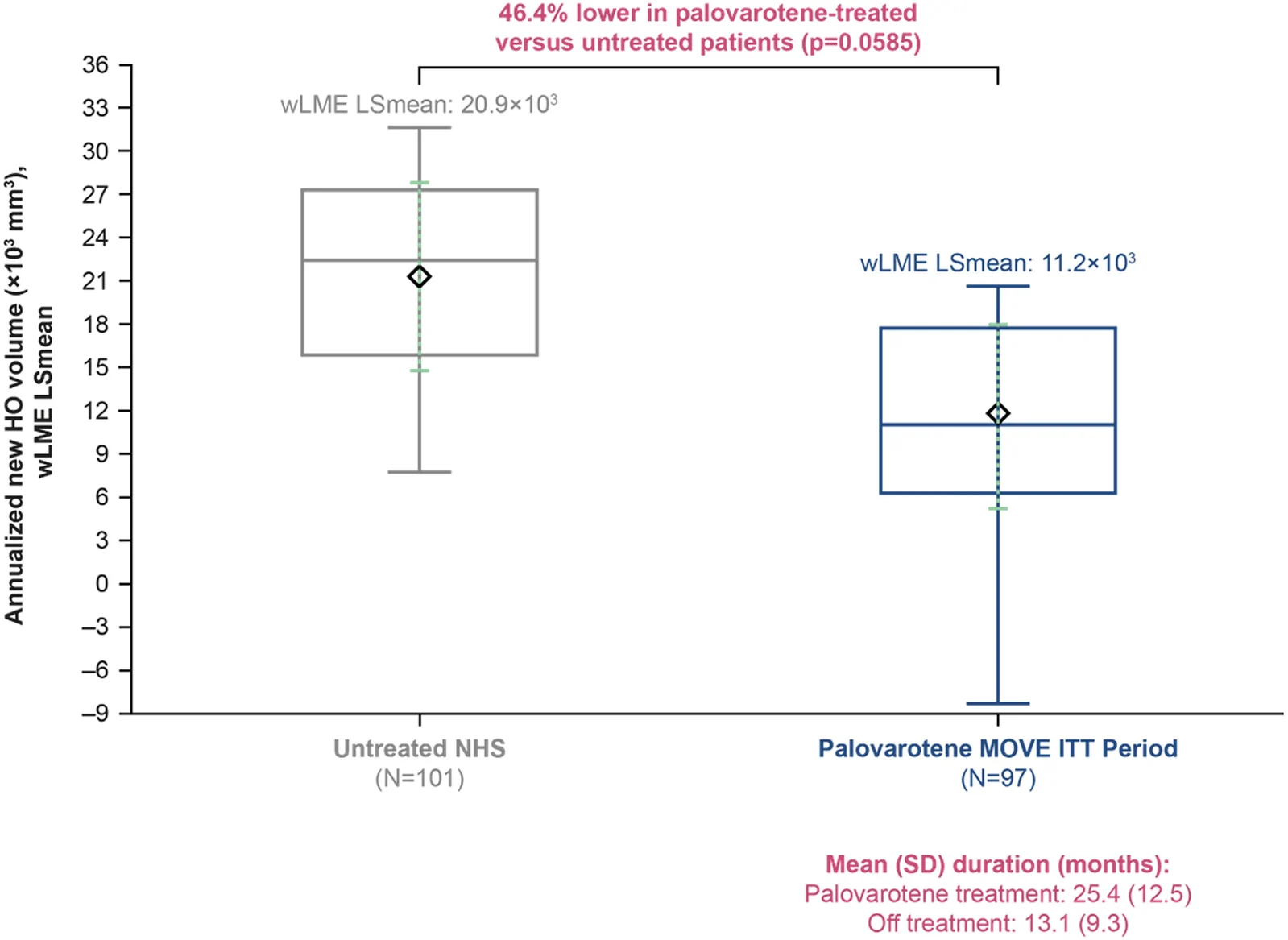
Annualized new HO volume from baseline to trial completion in the MOVE ITT period for patients receiving palovarotene compared with untreated patients from the NHS. Data presented are wLME LSmean (diamond), median (middle horizontal line), upper and lower quartile (box limits), min and max (box whiskers), and SD (green dotted error bars). Baseline total HO, baseline age, sex, baseline months since last flare-up, and baseline CAJIS were included as covariates in the wLME model. CAJIS, Cumulative Analogue Joint Involvement Scale; HO, heterotopic ossification; ITT, intent-to-treat; LSmean, least squares mean; max, maximum; min, minimum; NHS, natural history study; wLME, weight linear mixed-effect.

**Figure 3:**
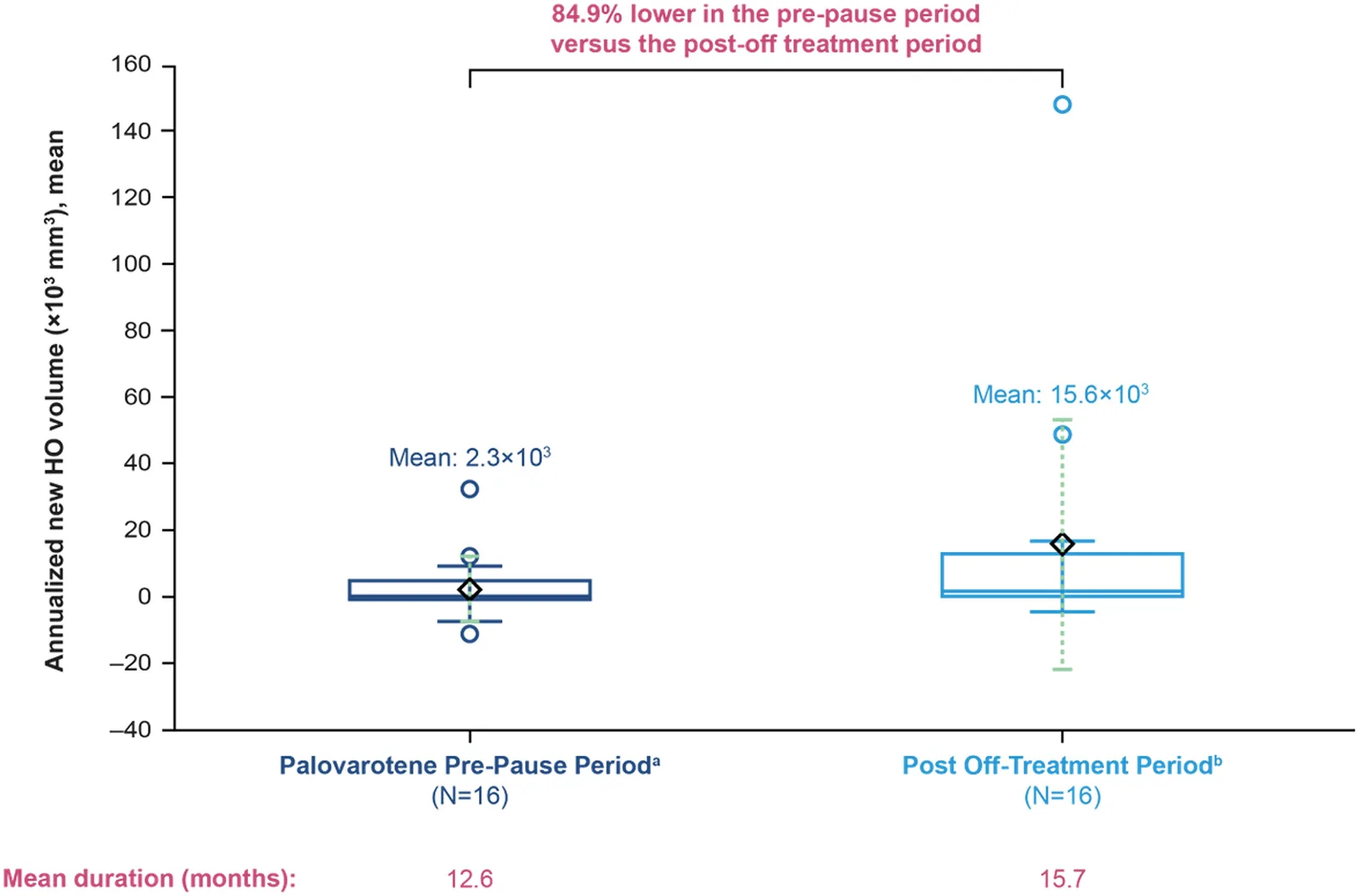
Annualized new HO volume from baseline to trial completion in the MOVE ITT period for patients who paused and stopped palovarotene treatment. Data presented are raw mean (diamond), median (middle horizontal line), upper and lower quartile (box limits), min and max (box whiskers), outliers (open circles), and SD (green dotted error bars). ^a^Defined as the period from the first WBCT scan to the last WBCT scan before treatment disruption. ^b^Spans the period from first WBCT scan off-treatment following dosing interruption, in patients who remained off treatment. HO, heterotopic ossification; ITT, intent-to-treat; max, maximum; min, minimum; NHS, natural history study; WBCT: whole-body CT.

**Figure 4:**
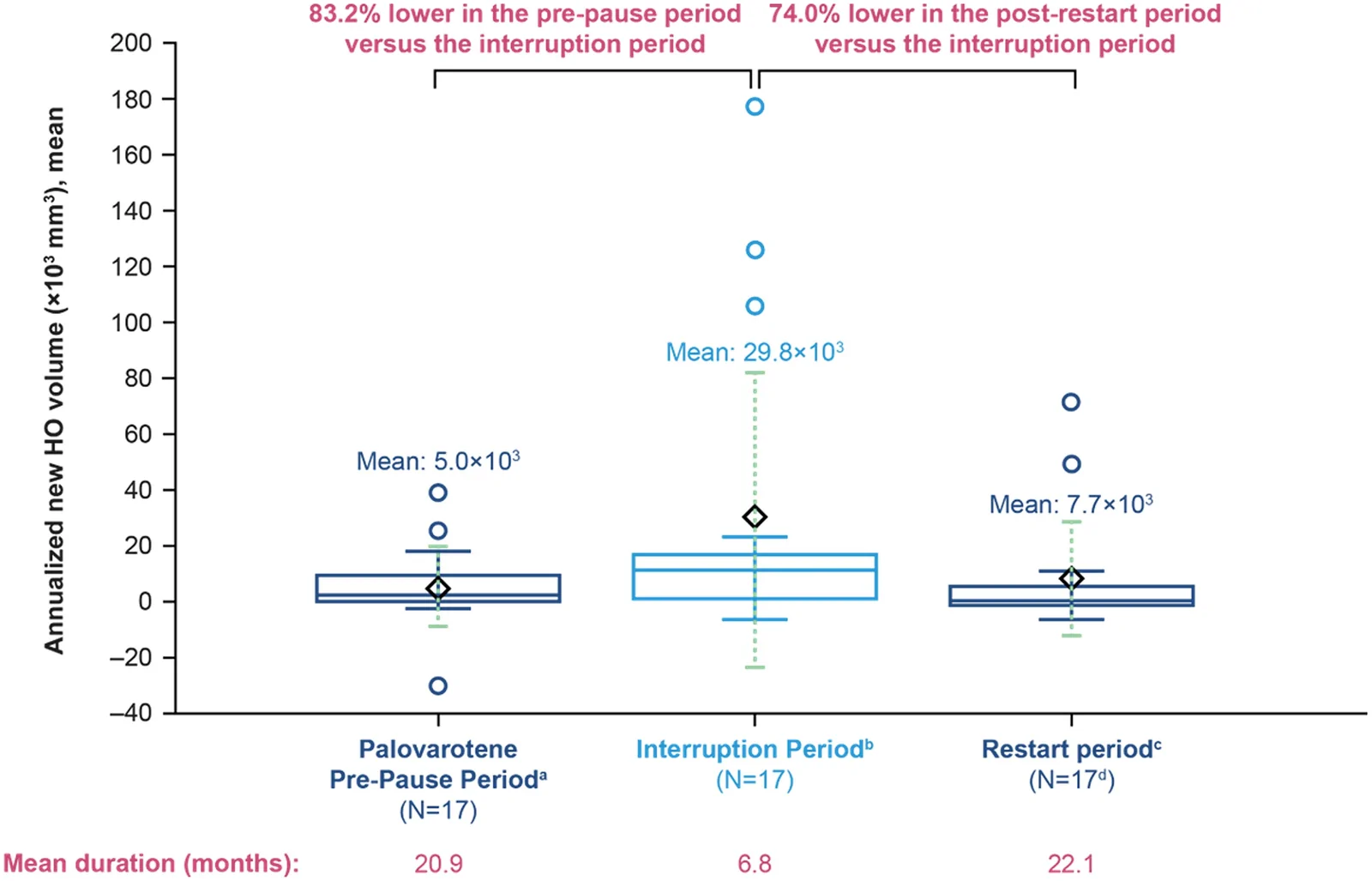
Annualized new HO volume from baseline to trial completion in the MOVE ITT period for patients who paused and restarted palovarotene treatment. Data presented are raw mean (diamond), median (middle horizontal line), upper and lower quartile (box limits), min and max (box whiskers), outliers (open circles), and SD (green dotted error bars). ^a^Defined as the period from the first WBCT scan to the last WBCT scan before treatment disruption. ^b^Spans the interruption due to the partial clinical hold and crossing the futility boundary, in patients who restarted palovarotene. ^c^Spans the time spent on treatment following interruptions with ≥2 WBCT scans post-restart. ^d^Limited patients paused and restarted palovarotene treatment due to missed WBCT scans and enrolment loss. HO, heterotopic ossification; ITT, intent-to-treat; max, maximum; min, minimum; WBCT: whole-body CT.

Final analyses from MOVE, across the entire 48-mo period, including time both on and off treatment, showed wLME least squares mean annualized new HO volume was 11.2 × 10^3^ mm^3^ (*n* = 97) in patients who received palovarotene in the MOVE ITT period and 20.9 × 10^3^ mm^3^ (*n* = 101) in untreated patients from the NHS (*p* = .0585; Figure 2). For the 16 palovarotene-treated patients in MOVE who paused and then subsequently stopped palovarotene treatment, mean annualized new HO volume was 2.3 × 10^3^ mm^3^ when patients received palovarotene and 15.6 × 10^3^ mm^3^ when they did not (84.9% difference; statistical significance not assessed; Figure 3). When patients stopped treatment, mean annualized new HO volume did not exceed that of untreated patients from the NHS. For the 17 palovarotene-treated patients in MOVE who paused and restarted treatment during the ITT period, mean annualized new HO volume was 5.0﻿ × ﻿10^3^ mm^3^ in the pre-pause period and 7.7 × 10^3^ mm^3^ in the post-restart period, and 29.8 × 10^3^ mm^3^ in the interruption period during which they did not receive palovarotene (83.2% and 74.0% difference, respectively; statistical significance not assessed; Figure 4).

### Secondary efficacy outcomes in MOVE

The proportion of patients who developed any new HO at the last timepoint assessed was 83.5% (81/97) in palovarotene-treated patients in the MOVE ITT period, which included time both on and off treatment, and was 75.2% (76/101) in untreated patients from the NHS. The proportion of patients in MOVE who developed any new HO was 81.0% (34/42) during the interruption period when patients did not receive palovarotene. Of patients who paused and restarted treatment or who paused and stopped treatment, 70.6% (12/17) and 75.0% (12/16) developed any new HO, respectively (statistical significance not assessed; Table S2). Patients receiving palovarotene in MOVE experienced a mean (SD) of 3.0 (1.7) body regions with new HO and this was 2.9 (1.7) in untreated patients from the NHS. The number of body regions with new HO across periods in MOVE is reported in Table S3 (statistical significance not assessed).

Mean (SD) flare-up rate per patient-month of exposure was 0.2 (0.4) in patients receiving palovarotene in the MOVE ITT period and 0.1 (0.1) in untreated patients from the NHS. During the interruption period in MOVE, mean (SD) flare-up rate was 0.1 (0.2), which did not change when patients restarted palovarotene treatment (statistical significance not assessed; Table S4).

### Exploratory efficacy outcomes in MOVE

At baseline, mean (SD) CAJIS score was 11.9 (7.3) in patients in the NHS and 9.8 (5.9) in patients in MOVE. In patients with a baseline and ≥1 post-baseline CAJIS score, the median duration of CAJIS follow-up from baseline to last visit was 42.2 mo in MOVE, with a mean increase from baseline of 1.8 (3.1) and a CAJIS score at last visit of 11.5 (6.4). The median duration of follow up was 25.4 mo in the NHS, mean increase from baseline to last visit was 1.0 (2.7), and CAJIS score at last visit was 13.0 (7.4; statistical significance not assessed; Table S5). Mean (SD) FOP-PFQ percentage of worst score was 46.6 (28.9) in patients in the NHS and 43.7 (26.1) in patients in MOVE at baseline. In patients with a baseline and ≥1 post-baseline record, the median duration of FOP-PFQ follow-up from baseline to last visit was 35.2 mo in MOVE, with a mean (SD) increase from baseline to last visit of 6.8 (14.9), and FOP-PFQ score at last visit of 50.5 (28.8). The median duration of follow-up was 22.9 mo in the NHS, mean (SD) increase from baseline to last visit was 6.2 (11.9), and FOP-PFQ score at last visit was 52.6 (28.7; statistical significance not assessed; Table S6).

Small decreases from baseline to last visit in PROMIS scores were seen in palovarotene-treated patients who had a baseline and ≥1 post-baseline record, and the results were generally comparable to those reported in untreated patients from the NHS (data not shown).

### Safety outcomes

Final safety analyses presented here include 107 patients from MOVE. At data cut-off, 66.4% of patients had palovarotene exposure for >30 mo. Overall mean (SD) duration of palovarotene dosing was 103.3 (54.0) wk; 75.7% (81/107) patients received flare-up treatment with mean (SD) duration of 34.7 (26.68) wk (Table S7).

All patients in MOVE reported at least one TEAE and 99.1% (106/107) reported at least one treatment-related TEAE (Table 1). The maximum treatment-related TEAE severity experienced was mild in 33.6% (36/107) of patients, moderate in 50.5% (54/107), and severe in 15.0% (16/107; Table 1). Commonly reported TEAEs included skin and s.c. tissue disorders, such as dry skin, dry lips, alopecia, drug eruption, and pruritus, and musculoskeletal disorders, such as arthralgia (Table 1). Incidences of TEAEs were generally similar between chronic and flare-up treatment, though a higher proportion of patients reported treatment-emergent SAEs with chronic treatment (31.8%; 34/107) vs flare-up treatment (23.5%; 19/81; Table 1).

**Table 1:** Treatment-emergent adverse events in palovarotene-treated patients through month 48 of MOVE.

***n* (%)**	**Chronic treatment (*N* = 107)**	**Flare-up treatment (*N* = 81)**	**Overall (*N* = 107)**
**Any TEAE**	105 (98.1)	77 (95.1)	107 (100.0)
**Any severe TEAE**	19 (17.8)	13 (16.0)	26 (24.3)
**Treatment-related TEAE**	98 (91.6)	75 (92.6)	106 (99.1)
Mild	51 (47.7)	27 (33.3)	36 (33.6)
Moderate	39 (36.4)	39 (48.1)	54 (50.5)
Severe	8 (7.5)	9 (11.1)	16 (15.0)
**Any serious TEAE**	34 (31.8)	19 (23.5)	48 (44.9)
**TEAE leading to dose modification**	11 (10.3)	32 (39.5)	38 (35.5)
**TEAE leading to dose interruption**	25 (23.4)	16 (19.8)	37 (34.6)
**TEAE leading to permanent discontinuation from palovarotene**	8 (7.5)	3 (3.7)	11 (10.3)
**TEAE leading to permanent discontinuation from the trial**	2 (1.9)	1 (1.2)	3 (2.8)
**Most common TEAEs** ^**a**^			
**Skin and subcutaneous tissue disorders**	91 (85.0)	69 (85.2)	105 (98.1)
Dry skin	60 (56.1)	37 (45.7)	76 (71.0)
Alopecia	19 (17.8)	21 (25.9)	38 (35.5)
Drug eruption	15 (14.0)	26 (32.1)	34 (31.8)
Pruritus	19 (17.8)	14 (17.3)	31 (29.0)
Rash	25 (23.4)	11 (13.6)	29 (27.1)
Erythema	11 (10.3)	16 (19.8)	26 (24.3)
Pruritus generalized	16 (15.0)	11 (13.6)	24 (22.4)
Skin exfoliation	11 (10.3)	11 (13.6)	21 (19.6)
Skin irritation	6 (5.6)	5 (6.2)	11 (10.3)
**Gastrointestinal disorders**	71 (66.4)	40 (49.4)	88 (82.2)
Lip dry	37 (34.6)	17 (21.0)	52 (48.6)
Chapped lips	6 (5.6)	10 (12.3)	15 (14.0)
Vomiting	11 (10.3)	5 (6.2)	15 (14.0)
Nausea	9 (8.4)	3 (3.7)	12 (11.2)
**Infections and infestations**	70 (65.4)	41 (50.6)	85 (79.4)
Upper respiratory tract infection	23 (21.5)	6 (7.4)	26 (24.3)
Nasopharyngitis	14 (13.1)	9 (11.1)	19 (17.8)
Paronychia	9 (8.4)	10 (12.3)	17 (15.9)
Corona virus infection	12 (11.2)	2 (2.5)	14 (13.1)
Influenza	7 (6.5)	4 (4.9)	11 (10.3)
**Musculoskeletal and connective tissue disorders**	65 (60.7)	37 (45.7)	80 (74.8)
Arthralgia	32 (29.9)	15 (18.5)	41 (38.3)
Pain in extremity	22 (20.6)	9 (11.1)	28 (26.2)
Epiphyses premature fusion	11 (10.3)	10 (12.3)	21 (19.6)
Musculoskeletal pain	7 (6.5)	5 (6.2)	12 (11.2)
Back pain	8 (7.5)	4 (4.9)	11 (10.3)
**Injury, poisoning and procedural complications**	53 (49.5)	28 (34.6)	67 (62.6)
Fall	10 (9.3)	7 (8.6)	16 (15.0)
Skin abrasion	8 (7.5)	9 (11.1)	15 (14.0)
Contusion	9 (8.4)	6 (7.4)	14 (13.1)
**Respiratory, thoracic and mediastinal disorders**	26 (24.3)	13 (16.0)	36 (33.6)
Cough	7 (6.5)	5 (6.2)	11 (10.3)
**Investigations**	25 (23.4)	17 (21.0)	34 (31.8)
Bone density decreased	8 (7.5)	5 (6.2)	13 (12.1)
**Eye disorders**	15 (14.0)	17 (21.0)	26 (24.3)
Dry eye	8 (7.5)	11 (13.6)	18 (16.8)
**Nervous system disorders**	16 (15.0)	10 (12.3)	24 (22.4)
Headache	10 (9.3)	7 (8.6)	16 (15.0)

Reported as *n* (%). ^a^TEAEs observed in ≥10% of patients in the overall group are reported. Abbreviations: AE, adverse event; TEAE, treatment-emergent AE.

Dose modifications were used to manage TEAEs in 35.5% (38/107) of patients, while dose interruptions occurred in 34.6% (37/107) of patients (Table 1), most commonly due to skin AEs (12/107; 11.2%). Overall, 10.3% (11/107) of patients experienced TEAEs leading to permanent discontinuation of palovarotene, most commonly due to musculoskeletal and connective tissue disorders, including epiphyses premature fusion (4/107; 3.7%; otherwise referred to as PPC; Table S8), and 2.8% (3/107) experienced TEAEs leading to permanent discontinuation from the trial (2 cases of PPC and 1 case of self-injury; Table 1).

Almost half of patients (44.9% [48/107]) experienced treatment-emergent SAEs, most commonly PPC (19.6% [21/107]) and corona virus infection (11.2% [12/107]). There were minimal differences in incidence of treatment-emergent SAEs between patients aged <18 yr and ≥18 yr, except for PPC.

### Incidence and management of skin AEs in MOVE

Post hoc analyses of the incidence and management of skin AEs were conducted for 97 patients from MOVE with at least one skin TEAE. Of these, 20.6% (20/97) had a history of skin and s.c. tissue disorders. The most common skin TEAEs were dry skin, dry lips, alopecia, drug eruption, pruritus, rash, erythema, pruritus generalized, skin exfoliation, and skin irritation (Figure S2).

Prophylactic therapies used to manage skin TEAEs and the mean duration of use are illustrated in Figure S3. The most common skin prophylaxis concomitant medication was soft paraffin and fat products (23.7% [23/97]), with a mean (SD) duration of treatment of 850.5 (607.9) d. The most frequent types of concomitant medications taken within 3 d of skin TEAE onset were other emollients and protectives (37.1% [36/97]), soft paraffin and fat products (26.8% [26/97]), prednisone (25.8% [25/97]), hydrocortisone (24.7% [24/97]), dimeticone (10.3% [10/97]), and triamcinolone (10.3% [10/97]; Table S9). Table S10 reports the predominant concomitant medications taken for the most commonly reported skin TEAEs.

Prophylaxis generally shortened the median duration of the most common skin TEAEs in patients receiving chronic and flare-up treatment vs without prophylaxis; this was not the case for drug eruption, rash, and skin irritation with chronic treatment, and dry skin and erythema with flare-up treatment (Figure S4). Skin TEAEs were mostly mild or moderate for both chronic and flare-up treatment; in patients receiving flare-up treatment, prophylaxis generally reduced TEAE intensity (Figure S5). Skin TEAEs led to palovarotene dose modification in 29.9% (29/97) and withdrawal in 2.1% (2/97) of patients. There were minimal differences in dosing adjustments made to manage skin TEAEs between chronic and flare-up treatment, and with vs without prophylaxis (Table S11).

### P‌PC in MOVE and the phase II OLE

**Figure 5:**
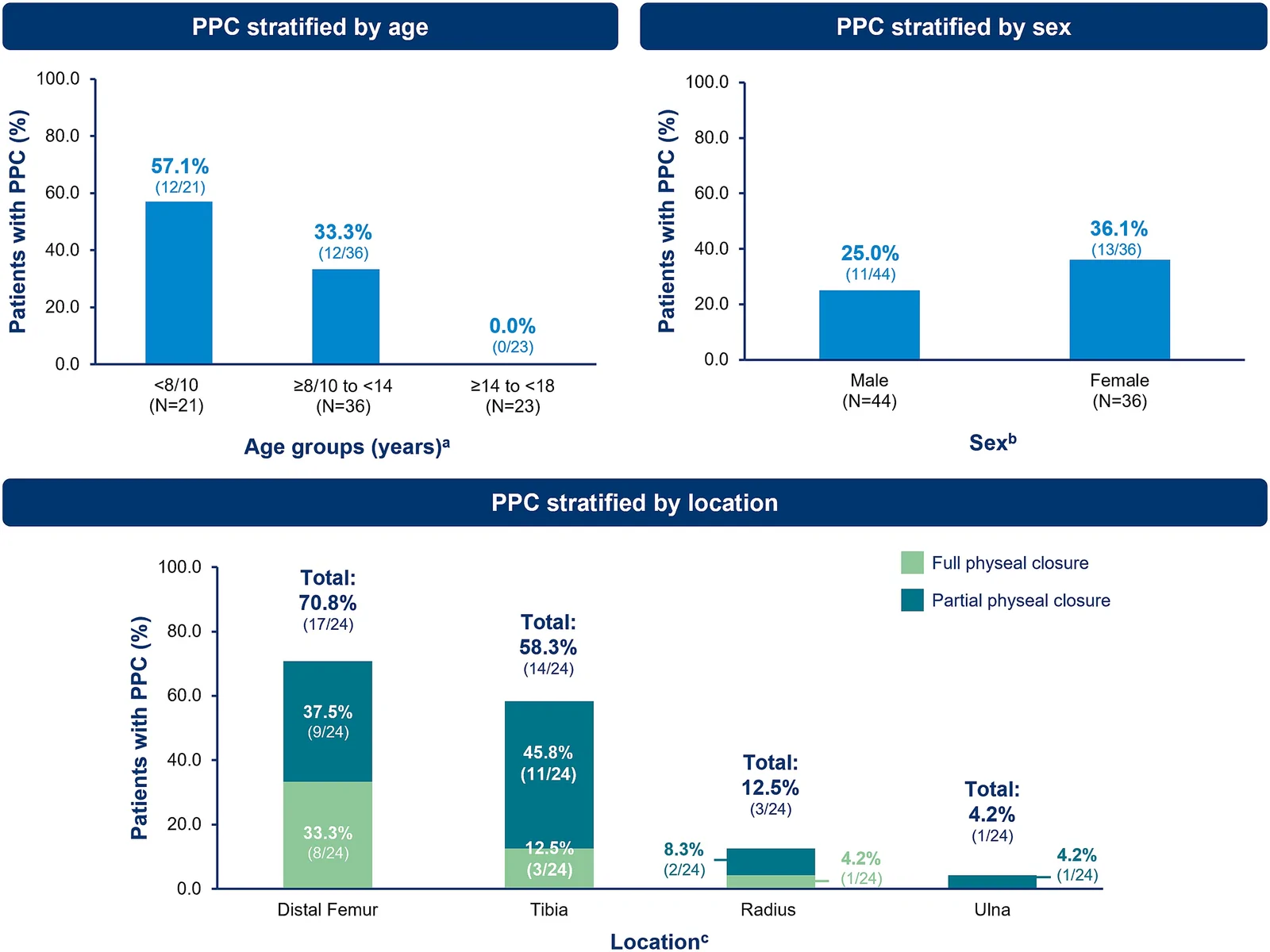
Incidence of PPC events in patients aged <18 yr in MOVE by age group, sex, and location. ^a^Ages are reported as female/male and patients were grouped by age at enrolment; *n*/*N* is the number of patients with PPC/total number of patients in each age group. ^b^*n*/*N* is the number of patients with PPC/total number of patients by sex. ^c^Includes patients with a PPC event status of closed or partially closed; *n*/*N* is the number of patients with PPC at each location/total number of patients with PPC. OLE, open-label extension; PPC, premature physeal closure.

In MOVE, the 80 patients aged <18 yr (age range: 4-17 yr) received palovarotene for a mean (SD) of 166.0 (75.2) wk. Of these, 24/80 (30.0%) had clinical events consistent with PPC, all of which occurred in those aged <14 yr (Figure 5); 3/24 cases occurred post-treatment. PPC was most commonly detected at the distal femur followed by the tibia, radius, and ulna, and events were more often partial rather than full closures (Figure 5). In the Phase II OLE, clinical events consistent with PPC were detected in 3/22 (13.6%) patients aged <18 yr, all of whom were aged <14 yr (Figure S6).

Of 13 patients aged ≥8 yr for females and ≥10 yr for males with PPC in MOVE and the phase II OLE, nine continued to grow after PPC diagnosis, 2 patients achieved near adult height at palovarotene initiation, 1 patient showed growth deceleration prior to palovarotene initiation, and 1 patient developed moderate scoliosis by month 12.

Of the patients aged ≥8 yr for females and ≥10 yr for males with PPC, 6 patients achieved a height within the normal adult range (equal to or above the fifth percentile) by the last follow-up visit (average height *z*-score at last visit: 0.7). Of these, 3 had moderate scoliosis that contributed to height deceleration, 2 did not exhibit detrimental effects on growth, and 1 was near adult height at palovarotene initiation. Seven patients had heights below the fifth percentile for sex-matched peers by the last follow-up visit (average height *z*-score at last visit: −1.7). Of these, 5 had full closure at the distal femur, 3 patients showed growth deceleration prior to palovarotene initiation, and 6 patients showed growth deceleration after palovarotene initiation (1 was near adult height at the time of palovarotene; 5 had moderate scoliosis and/or severe kyphosis [not related to palovarotene] that contributed to growth deceleration). One patient achieved growth stabilization following discontinuation of palovarotene.

None of the 13 patients aged ≥8 yr for females and ≥10 yr for males with PPC demonstrated medically significant leg length discrepancy or a distal femoral angle that would indicate angular deformity from baseline up to the last follow-up visit.

### Biomechanical CT analyses in MOVE and the phase II OLE

Biomechanical CT analyses were performed for 107 patients from MOVE and 54 patients from the phase II OLE who received palovarotene and were compared with 114 untreated patients from the NHS. The mean (SD) age was 15.5 (10.1) yr in MOVE, 21.0 (9.2) yr in the phase II OLE, and 17.6 (9.7) yr in the NHS (Table S12). Mean follow-up length was 32.7 mo in MOVE, 42.7 mo in the phase II OLE, and 25.9 mo in the NHS.

After adjustment for baseline characteristics, association between palovarotene treatment and continuous spinal health outcomes are presented for MOVE (Table 2) and the phase II OLE (Table S13). In MOVE and the phase II OLE, palovarotene was associated with 0.55 mg/cm^3^ (0.52%) and 0.04 mg/cm^3^ (0.04%) losses in calculated BMD, 0.01 g (0.24%) and 0.15 g (2.94%) losses in calculated BMC, and a 1.23 (0.03%) newton increase and 180.18 (3.83%) newton decrease in calculated vertebral body strength for each year from baseline, respectively.

**Table 2:** Adjusted regression model calculated change in vertebral body strength, entire vertebral body BMC and mid-vertebral (trabecular) density per year by effect in patients receiving palovarotene in MOVE (*N* = 85) vs untreated patients from the NHS (*N* = 85).

	**Mid-vertebral (trabecular) density, mg/cm** ^**3**^	**Entire vertebral body BMC, g**	**Vertebral body strength, newton**
**Sex, female**	2.49^a^ (0.55, 4.43)	−0.04 (−0.16, 0.09)	48.47 (−76.51, 173.45)
**Age at FOP symptom onset**	0.03 (−0.17, 0.24)	−0.01 (−0.02, 0.01)	−9.48 (−27.33, 8.36)
**Steroid use**	−1.20 (−3.13, 0.73)	−0.05 (−0.18, 0.07)	−77.41 (−208.38, 53.56)
**Baseline CAJIS score**	−0.12 (−0.35, 0.11)	−0.02^a^ (−0.03, 0.00)	−13.30^a^ (−26.05, −0.55)
**Palovarotene treatment**	−0.55 (−2.30, 1.20)	−0.01 (−0.09, 0.07)	1.23 (−85.67, 88.13)

Data are presented as beta (95% CI). ^a^*p* < .05. CAJIS, cumulative analogue joint involvement scale; FOP, fibrodysplasia ossificans progressiva; NHS, natural history study.

In MOVE, patients receiving palovarotene had a mean (SD) of 0.3 (0.7) new-onset, computationally detected vertebral deformities/fractures annually (*N* = 91), while patients in the NHS had 0.1 (0.3) annually (*N* = 92). Patients receiving palovarotene in MOVE had a 2.29 (95% CI: 1.23-4.26) times significantly higher risk of computationally detected, radiographically observed, asymptomatic, annualized new-onset vertebral deformities/fractures compared with untreated patients from the NHS (*p*  <0.01). The annualized risk of computationally detected, moderate/severe new-onset vertebral deformities/fractures in MOVE compared with the NHS was not statistically significant (2.59; 95% CI: 0.84-8.00). In the phase II OLE, there was no statistically significantly higher risk of either computationally detected new-onset or moderate/severe new-onset vertebral deformities/fractures in palovarotene-treated patients compared with patients from the NHS.

## Discussion

Palovarotene has been approved for the reduction of new HO in adult and pediatric patients with FOP aged ≥8 yr (females) and ≥10 yr (males) in several countries globally,^16–19^^,^^21^ based on post hoc interim analyses from month 18 (IA3) of the phase III MOVE trial, which provided the most complete dataset from the trial.^24^ We present exploratory post hoc analyses from the entire 48-mo MOVE trial, supplemented by data from the phase II OLE, to provide supportive longer-term efficacy and safety data. Due to the rarity of FOP, the NHS provided a comparator group of untreated patients for these analyses.^9^^,^^29^

Mean annualized new HO volume, including HO formation from new and existing lesions, was numerically (46.4%) lower in palovarotene-treated patients from the 48-mo MOVE ITT period vs the NHS. While the numerical decrease observed may support previous findings from MOVE,^24^ this difference did not reach statistical significance and was not adjusted for multiple comparisons. Treatment interruptions allowed comparisons of periods on and off palovarotene treatment within MOVE; mean annualized new HO volume appeared lower during treatment and no evidence of rebound was observed, but statistical significance was not assessed. Given the exploratory nature of these analyses, small sample size, and treatment interruptions, results should overall be interpreted with caution and definitive conclusions cannot be drawn as the findings may be due to chance. It should also be noted that the HO volume analyses presented here were not adjusted for corticosteroid use, which were used by a majority of patients in MOVE. Corticosteroids are often used in patients with FOP in the management of flare-ups to reduce inflammation; for example, 71.2% of flare-ups in the NHS were treated with corticosteroids.^9^ However, progressive accumulation of new HO in patients is observed despite the use of corticosteroids, and therefore, there is no evidence supporting a corticosteroid-related disease-modifying effect.^8^^,^^9^

Secondary outcomes (ie, new HO incidence, number of body regions affected) were similar in patients who received palovarotene in MOVE and untreated patients from the NHS. This contrasted with results suggesting a numerically lower mean annualized new HO volume in treated vs untreated patients. Currently, there is no consensus on a threshold for a clinically meaningful reduction in new HO volume in patients with FOP, and this warrants further research. However, analyses of data from the NHS showed that higher mean HO volumes were associated with reduced motion in all body regions, and higher mean HO volumes in specific body regions were associated with worse joint-specific CAJIS scores within those regions.^9^ Since a pathognomonic feature of FOP is cumulative and irreversible HO,^6^^,^^7^ reducing the volume of new HO over time may help slow the progression of functional disability, and preventing even small volumes of new HO in certain joints may be crucial for maintaining patients’ mobility and quality of life.^9^^,^^12^^,^^29^ Future analyses could investigate new HO volume in individual joints and body regions; indeed, joint ankyloses are largely driven by HO in and around the shoulders, elbows, hips, and knees, so assessing total HO volume may overestimate the contribution of axial HO to joint ankyloses. Analyses may thus be improved by adjusting for axial HO volume; however, the implications of axial HO on the progression of thoracic deformity, thoracic insufficiency, and early mortality must be considered.^30^

Across the MOVE ITT period, mean flare-up rate per patient-month of exposure was numerically greater in palovarotene-treated patients than in untreated patients from the NHS, although statistical significance was not assessed, and the rates were overall not notably different from published literature.^8^^,^^31^ The observed difference compared with the NHS may result from the more frequent clinical contact during MOVE.

Exploratory functional endpoints (ie, CAJIS and FOP-PFQ) showed small increases in MOVE. However, it should be recognized that there were challenges associated with using CAJIS and FOP-PFQ within these clinical trials due to lack of annualization, the fact that these outcomes are unlikely to be sensitive enough to detect changes in the limited time frames of the trials, and statistical significance not being assessed; therefore, robust conclusions cannot be drawn. Additionally, clinically meaningful changes in CAJIS and FOP-FPQ have not been defined, and these may depend on specific joint location for CAJIS and the treatment goals of individual patients.

The AE profile of palovarotene remained consistent with previous findings and with other systemic retinoids, consisting mainly of mild-to-moderate skin and s.c. tissue and musculoskeletal events.^23^^,^^24^^,^^32^ The International Clinical Council (ICC) on FOP recognize the impact of palovarotene treatment on the skin and it is recommended to manage skin AEs with prophylactic/symptomatic therapies and/or dose modifications.^14^^,^^33^ The U.S. FDA also acknowledges the need to prevent or treat skin AEs associated with palovarotene.^16^ Results presented here suggest that prophylactic therapies may reduce the intensity and duration of skin TEAEs, particularly in patients receiving flare-up palovarotene treatment. Prophylactic therapies have been found to improve treatment compliance rates in patients with other chronic diseases, and therefore may also increase the adherence of patients with FOP to palovarotene.^34^ Thus, prophylactic measures may help to manage skin AEs and improve adherence, though optimal strategies require further exploration.

PPC is a known risk associated with palovarotene treatment in skeletally immature patients.^24^ Consistent with other systemic retinoids, PPC events in MOVE predominantly affected patients’ lower limbs^35^; however, this occurred sooner and more frequently with palovarotene than the expected class effect.^36^^,^^37^ This apparent difference in the effect of palovarotene vs other systemic retinoids on PPC by body region in MOVE warrants further research. It should be noted that in MOVE, a conservative approach to the classification of PPC events was taken to ensure that all cases were recorded as SAEs, including all PPC events identified by the Investigator regardless of whether seriousness criteria were met. Nevertheless, in growing patients, there may be potentially irreversible risks of PPC, and any early signs of PPC should be assiduously heeded. Data presented here from MOVE and the phase II OLE showed that of the 13 patients aged ≥8 yr for females and ≥10 yr for males who experienced PPC events, 6 achieved normal adult height by the end of the study and 7 had a height below the fifth percentile. The impact of palovarotene on height in growing patients remains a serious concern. Although some patients achieved normal adult height, the wide variability observed, and the likelihood that these outcomes may differ from expected growth patterns, limits the interpretability of these findings. Should any evidence of PPC and/or adverse effects on growth be observed with palovarotene treatment, the potential risks should be weighed against the potential benefits of reducing new HO volume, to determine the appropriateness of continued treatment compared with temporary or permanent discontinuation. While early treatment of FOP to reduce irreversible HO may be beneficial for improving disease progression, the risk of PPC and potential impact on height with palovarotene may limit the ability to treat growing patients. Therefore, there remains a need for additional disease-modifying therapies to treat younger pediatric patients with FOP. Clinical trials to assess the efficacy and safety of other investigational treatments for FOP are ongoing, including in pediatric patients.

Computationally predicted biomechanical CT results presented here suggest that palovarotene treatment was associated with small changes in predicted spinal BMD, BMC, and bone strength in patients with FOP compared with untreated patients. Although these data showed that changes were of low magnitude and were highly variable across MOVE and the phase II OLE, we did not expect to see reductions in these outcomes in the natural progression of growing individuals; therefore, the clinical significance of these findings is uncertain and requires further investigation. Biomechanical CT is a novel computational method for providing non-invasive measurements of BMD, BMC, and bone strength; however, it has not been validated in patients with rare diseases, specifically FOP, in whom scoliosis, kyphosis, and axial HO may affect BMD and vertebral strength. Therefore, the full implications of applying this methodology in the analysis of FOP are currently unknown. Adjustments made for baseline characteristics did not take into account axial HO or spinal CAJIS scores, which may impact BMD and vertebral deformity. Furthermore, biomechanical CT has other limitations in clinical application; specifically, it cannot detect molecular level defects in bone that might compromise bone strength independently of the level of BMD, and different implementations of biomechanical CT can provide different values of bone strength depending on a number of technical factors. Although DXA is the established standard for measuring BMD, this approach is not possible in patients with FOP given the excess bone and ankylosis throughout the skeleton. Biomechanical CT predictions are not impacted by HO and, therefore, remain valuable in increasing the understanding of skeletal bone health in patients with FOP.

Radiographically observed, asymptomatic vertebral deformity was used as a surrogate measure of fracture risk and was 2.29 times higher in patients receiving palovarotene vs untreated patients, with this difference reaching statistical significance; this increased fracture risk was not observed in those receiving palovarotene in the phase II OLE. No clinically significant, symptomatic vertebral deformities (classified as fractures in this analysis) were reported in MOVE or the phase II OLE; however, the definition for clinically significant, symptomatic vertebral fractures was not provided in the MOVE trial protocol, and therefore potential fracture symptoms, such as pain, could have been misrecorded as flare-ups. As deformities/fractures were defined computationally here, it cannot be confidently concluded that the vertebral morphological deformities observed were true spinal compression fractures, due to observed changes in FOP-related vertebral structure.^38^ Nevertheless, the significantly increased incidence of radiographically observed, asymptomatic vertebral deformities could be predictive of future fracture risk, particularly given the unexpected small decreases in spinal BMD, BMC, and bone strength, and therefore the potential impact on patients must be considered when determining appropriateness of palovarotene treatment.

Overall, long-term, exploratory, post hoc efficacy analyses from the entire 48-mo period of MOVE suggested that new HO volume may be lower in palovarotene-treated patients compared with untreated patients from the NHS. The rarity of FOP, along with the treatment interruptions that occurred during MOVE, limited the sample size available for analyses; however, beyond IA3, analyses utilizing data from periods within MOVE when patients were untreated as a comparator were supportive of the findings at month 18.^24^ Nevertheless, given these limitations and the fact that statistical analyses were not performed, results should be treated with caution and definitive conclusions should not be drawn as the findings may be due to chance. Palovarotene treatment had an AE profile that was consistent with previous findings and other systemic retinoids; however, the potentially irreversible risks of PPC in growing patients, and subsequent impact on height, must be closely monitored and considered alongside the potential benefits of treatment. This may limit the ability to treat growing patients with palovarotene and thus highlights the need for additional disease-modifying therapies for these individuals. The assessment of real-world data in upcoming studies, such as the FOPal international, observational registry study (NCT06089616),^39^ will provide further information on the long-term efficacy and safety of palovarotene in patients with FOP.

## Supplementary Material

Palovarotene_Long_Term_Efficacy_and_Safety_Supplement_13Feb26_ziag141

## Data Availability

Qualified researchers with a valid research question may request anonymized patient-level data by contacting an Ipsen representative. Further information on Ipsen’s Data Sharing policy is available here (Clinical Data Transparency-Global).

## Medical writing support

The authors thank Erin Clarkson, BSc, and Beverley Wilson, PhD, of Costello Medical, UK, for providing medical writing support, which was sponsored by Ipsen in accordance with Good Publication Practice guidelines.
