# Supplementary material for: Palovarotene impact on fibrodysplasia ossificans progressiva (FOP): data from month 48 of the phase III MOVE trial
Source: JBMR Plus. 2026 Aug 29;10(10):ziag141. doi: 10.1093/jbmrpl/ziag141 (PMC13592773; doi:10.1093/jbmrpl/ziag141)
Supplement: Supplementary file 1 [file palovarotene_long_term_efficacy_and_safety_supplement_13feb26_ziag141.docx]

**SUPPLEMENTARY APPENDIX**

1. SUPPLEMENTARY METHODS

## 1.1 Phase II OLE: Patients and Trial Design

The phase II open-label extension (OLE) trial (NCT02279095) was an extension of the randomized, double-blind, placebo-controlled, multicenter phase II trial (NCT02190747), and evaluated the efficacy and safety of various palovarotene dosing regimens.^1^ Patients with clinically diagnosed FOP with the *ALK2/ACVR1*^R206H^ pathogenic variant who were enrolled in the original phase II trial were eligible to enroll in the OLE.^1^ The trial was divided into four parts.^1^ In Part A, palovarotene 10/5 mg flare-up treatment (10 mg for 2 weeks followed by 5 mg for 4 weeks) was administered in participants reporting a qualifying flare-up. In Part B, palovarotene 20/10 mg flare-up treatment (20 mg for 4 weeks followed by 10 mg for 8 weeks) was administered in participants reporting a qualifying flare-up, and palovarotene 5 mg chronic treatment was administered daily for up to 24 months in skeletally mature participants. In Part C, all patients received palovarotene 5 mg chronic treatment for up to 36 months, and the 20/10 mg flare-up treatment in the event of a qualifying flare-up. Part D was added for skeletally immature patients who stopped taking palovarotene for any reason before completion of Part C. Part D included yearly visits for an up to 2-year follow-up period following the last dose. No palovarotene dosing occurred during Part D. Assessments in Part D included yearly linear height, knee height, weight, physical exams, vital signs, radiographic assessments of the knee and hand/wrist, low-dose whole-body computed tomography (WBCT) scans (excluding head), AEs, and concomitant medications. Participation in Part D continued as long as patients remain skeletally immature, and the up to 2-year period began the last day the patient stopped receiving palovarotene in Part C. The total duration of participation in Part C and Part D was a maximum of 4 years.

## 1.2 Sensitivity Analyses

The following post hoc sensitivity analyses were performed for weighted linear mixed-effects (wLME) mean annualized HO volume changes at MOVE IA3: analysis with additional covariates (including baseline HO volume divided by baseline age, sex, baseline months since last flare up, baseline CAJIS, and treatment), analysis with propensity score quartile as a covariate, analysis with extreme annualized new HO volumes of >100.0×10^3^ mm^3^ set to 100.0×10^3^ mm^3^, analysis of patients who transitioned from the NHS to MOVE, and propensity score matched and weighted analyses to adjust for baseline differences. A pre-specified analysis was also conducted using generalized estimating equations (GEE) modelling without weights.

2. SUPPLEMENTARY FIGURES

## Figure S1. Annualized new HO volume in palovarotene-treated and untreated patients at MOVE IA3 using GEE modelling without weights^a^

Data presented are GEE LSmean (diamond), median (middle horizontal line), upper and lower quartile (box limits), min and max (box whiskers), outliers (open circles), and SD (green dotted error bars).^a^Included annualized new HO as a dependent variable and fixed effects of treatment and baseline HO volume divided by baseline age as independent variables; ^b^Reductions in annualized new HO volume with palovarotene treatment were statistically significant compared with untreated patients. GEE: Generalized estimating equations; HO: heterotopic ossification; Lsmean: least-squared mean; SD: standard deviation.

## Figure S2. Most common skin TEAEs in patients receiving chronic and flare-up palovarotene treatment, with and without skin prophylaxis.

Skin TEAEs were defined by a “skin and subcutaneous tissue disorders” System Organ Class or a “lip dry” Preferred Term. Data are presented as percentage of patients (number of patients) [number of events]; percentages were calculated based on the total number of patients who received chronic (N=97) and flare-up (N=76) treatment, respectively. If a patient experienced more than one event in a category, the patient was counted only once in that category. Percentages were calculated with the number of patients in each treatment group as the denominator. ^a^Treatment emergence for chronic treatment was defined as any AE with an onset date on or after first dose date of chronic treatment and up to seven days past the date of last dose of chronic treatment; ^b^Treatment emergence for flare-up treatment was defined as any AE with an onset date on or after first dose date of flare-up treatment and up to seven days past the date of last dose of flare-up treatment; ^c^Drug eruption was the MedDRA Preferred Term for “retinoid dermatitis”, defined as a triad of generalised rash, dry skin and erythema with onset of all three findings within 2–3 days; ^d^Only TEAEs occurring while on skin concomitant medications taken as prophylaxis that started or were ongoing seven days prior to start date of TEAEs were included. Data for skin AEs in ≥10 patients are reported. Other reported skin AEs included dermatitis (E=10), ingrowing nail (E=13), rash generalized (E=13), rash maculo-papular (E=11), eczema (E=12), acne (E=6), dandruff (E=5), onycholysis (E=5), decubitus ulcer (E=18), skin ulcer (E=7), blister (E=5), skin disorder (E=4), urticaria (E=4), generalized erythema (E=4), skin pain (E=4), hidradenitis (E=3), rash pruritic (E=3), skin erosion (E=3), skin mass (E=3), swelling face (E=3), dermatitis acneiform (E=3), xeroderma (E=3), dermal cyst (E=2, hand dermatitis (E=2), madarosis (E=2), scab (E=2), skin fragility (E=2), skin hyperpigmentation (E=2), skin lesion (E=2), abnormal hair texture (E=2), cold sweat (E=1), dermatitis allergic (E=1), dermatosis (E=1), face oedema (E=1), granuloma skin (E=1), hyperhidrosis (E=1), hyperkeratosis (E=1), hypertrichosis (E=1), intertrigo (E=1), keratosis pilaris (E=1), mechanical urticaria (E=1), perioral dermatitis (E=1), petechiae (E=1), photosensitivity reaction (E=1), pityriasis rosea (E=1), post inflammatory pigmentation change (E=1), rash erythematous (E=1), rash macular (E=1), rash papular (E=1), sensitive skin (E=1), skin discolouration (E=1), skin discomfort (E=1), skin induration (E=1), skin odour abnormal (E=1), skin plaque (E=1), skin reaction (E=1), skin sensitization (E=1), solar dermatitis (E=1), sticky skin (E=1). TEAE: treatment-emergent adverse event.

## Figure S3. Most common skin prophylaxis concomitant medications prior to skin TEAE onset and duration of prophylactic treatment.

Data are presented as proportion of patients (mean [SD] duration in days of skin prophylaxis concomitant medications). Data are presented for prophylaxis concomitant medications used by greater than 1.0% of patients; prophylaxis concomitant medications used by 1.0% of patients included: Aquaphor healing, Aveeno bath, Curel, Epaderm, heparinoid, lipikar, Nivea, allantoin/camphor/oxybenzone/padim, Banana boat, Neutrogena ultra sheer, zinc oxide, aloe vera, Cocos nucifera oil, medicated shampoo, other dermatologicals, pyrithione zinc, certizine hydrochloride, hydroxyzine, triamcinolone, Ducray kelual DS, nystatin, biotin. All skin prophylaxis concomitant medications that started or were ongoing 7 days prior to start date of the skin TEAE of each patient were selected. Prophylaxis medications which started more than 7 days before first palovarotene intake were excluded. Prophylaxis medications which started the day of TEAE onset were not counted for that particular AE. SD: standard deviation; TEAE: treatment-emergent adverse event.

## Figure S4. Median TEAE duration in patients receiving chronic and flare-up palovarotene treatment, with and without skin prophylaxis.

Data are presented as median (IQR). Data for skin AEs in ≥10 patients are reported. IQR: interquartile range; TEAE: treatment-emergent adverse event.

## Figure S5. Skin TEAE intensity in patients receiving (A) chronic and (B) flare-up palovarotene treatment, with and without skin prophylaxis.

(A)

(B)

Data are presented as percentage of events (number of events) [total number of events]; percentages were calculated based on the total number of events. Skin TEAE intensity was graded using the Common Terminology Criteria for Adverse Events (version 4.03). Data for skin AEs in ≥10 patients are reported. TEAE: treatment-emergent adverse event.

## Figure S6. Incidence of PPC events in patients aged <18 years by age group, sex, and location in the phase II OLE.

^a^Ages are reported as female/male and patients were grouped by age at enrolment; n/N is the number of patients with PPC/total number of patients in each age group. ^b^n/N is the number of patients with PPC/total number of patients by sex. ^c^Includes patients with a PPC event status of closed or partially closed; n/N is the number of patients with PPC at each location/total number of patients with PPC. OLE: open-label extension; PPC: premature physeal closure.

3. SUPPLEMENTARY TABLES

## Table S1. wLME sensitivity analyses for reductions in annualized new HO volume in palovarotene-treated compared with untreated patients.

|  | **% reduction in annualized new HO volume (palovarotene versus untreated)** | **Nominal p value** | **Palovarotene (MOVE)** | **Untreated (NHS)** |
| --- | --- | --- | --- | --- |
| **MOVE IA3 analysis**^2^ | | | | |
| wLME analysis^a^ | 53.8 | 0.0392^f^ | N=97 | N=101 |
| **MOVE IA3 sensitivity analyses** | | | | |
| Analysis with additional covariates^b^ | 56.2 | 0.0314^f^ | N=97 | N=97 |
| Analysis with propensity score quartile as a covariate^c^ | 56.7 | 0.0264^f^ | N=97 | N=97 |
| Analysis with extreme HO volumes >100.0×10^3^ mm^3^ set to 100.0×10^3^ mm^3^ | 51.0 | 0.0103^f^ | N=97 | N=101 |
| Analysis of patients who transitioned from the NHS to MOVE^d^ | 51.6 | 0.0634 | N=39 | N=39 |
| Propensity score matched analysis^e^ | 76.9 | <0.05^f^ | N=39 | N=39 |
| Propensity score weighted analysis^e^ | 67.2 | <0.05^f^ | N=58 | N=61 |

^a^Included baseline HO volume divided by baseline age as a covariate. ^b^Included baseline HO volume divided by baseline age, baseline age, sex, baseline months since last flare-up, baseline CAJIS, and treatment as covariates. ^c^Included propensity score quartile as the only covariate. ^d^Included 39 patients who transitioned from the NHS to MOVE. ^e^Excluded patients who transitioned from the NHS to MOVE; propensity scores were estimated via a multivariable logistic regression based on baseline age, sex, age-adjusted baseline HO, baseline CAJIS, and time since last flare-up. ^f^Statistically significant reduction in mean annualized new HO volume with palovarotene treatment. HO: heterotopic ossification; IA3: interim analysis 3; NHS: natural history study; wLME: weighted linear mixed effects.

## Table S2. Proportion of patients receiving palovarotene in MOVE and untreated patients from the NHS with any new HO after 48 months.

|  | **MOVE** | **NHS** |
| --- | --- | --- |
| **ITT period**^a^  N  New HO, n (%)  Yes  No | 97  81 (83.5)  16 (16.5) | 101  76 (75.2)  25 (24.8) |
| **Interruption period^b^**  N  New HO, n (%)  Yes  No | 42  34 (81.0)  8 (19.0) | N/A  N/A  N/A |
| **Restart period**^c^  N  New HO, n (%)  Yes  No | 17  12 (70.6)  5 (29.4) | N/A  N/A  N/A |
| **Post off-treatment period**^d^  N  New HO, n (%)  Yes  No | 16  12 (75.0)  4 (25.0) | N/A  N/A  N/A |

Data are presented as n (%). Percentages were calculated from the total in each treatment population. ^a^Overall analyses encompassing time from screening through last WBCT scan; ^b^Spans the interruption due to the partial clinical hold and crossing the futility boundary, in patients who restarted palovarotene; ^c^Spans the time spent on treatment following interruptions with ≥2 WBCT scans post-restart; ^d^Spans the period from first WBCT scan off-treatment following dosing interruption, in patients who remained off treatment. HO: heterotopic ossification; ITT: intent-to treat; N/A: not applicable; NHS: natural history study.

## Table S3. Number of body regions with new HO in patients receiving palovarotene in MOVE and untreated patients from the NHS after 48 months.

|  | **MOVE**  **(N=107)** | **NHS**  **(N=117)** |
| --- | --- | --- |
| **ITT period**^a^  n  Mean (SD)  Median  Min, Max | 81  3.0 (1.7)  3.0  1.0, 8.0 | 76  2.9 (1.7)  2.0  1.0, 7.0 |
| **Interruption period**^b^  n  Mean (SD)  Median  Min, Max | 34  2.6 (1.3)  2.0  1.0, 6.0 | N/A  N/A  N/A  N/A |
| **Restart period**^c^  n  Mean (SD)  Median  Min, Max | 12  3.3 (1.3)  3.5  1.0, 5.0 | N/A  N/A  N/A  N/A |
| **Post off-treatment period**^d^  n  Mean (SD)  Median  Min, Max | 12  2.3 (1.5)  2.0  1.0, 6.0 | N/A  N/A  N/A  N/A |

^a^Overall analyses encompassing time from screening through last WBCT scan; ^b^Spans the interruption due to the partial clinical hold and crossing the futility boundary, in patients who restarted palovarotene; ^c^Spans the time spent on treatment following interruptions with ≥2 WBCT scans post-restart; ^d^Spans the period from first WBCT scan off-treatment following dosing interruption, in patients who remained off treatment. N/A: not applicable; max: maximum; min: minimum; NHS: natural history study; SD: standard deviation.

## Table S4. Flare-up rates per patient-month of exposure in patients receiving palovarotene in MOVE compared with untreated patients from the NHS.

|  | **MOVE**  **(N=107)** | **NHS**  **(N=117)** |
| --- | --- | --- |
| **ITT period**^a^  n  Mean (SD)  Median  Min, Max | 107  0.2 (0.4)  0.1  0.0, 2.3 | 117  0.1 (0.1)  0.0  0.0, 0.9 |
| **Interruption period**^b^  n  Mean (SD)  Median  Min, Max | 49   0.1 (0.19)   0.0  0.0, 0.9 | N/A  N/A  N/A  N/A |
| **Post-restart period**^c^  n  Mean (SD)  Median  Min, Max | 49   0.1 (0.12)   0.0  0.0, 0.5 | N/A  N/A  N/A  N/A |
| **Post off-treatment period**^d^  n  Mean (SD)  Median  Min, Max | 58   0.1 (0.28)   0.0  0.0, 1.9 | N/A  N/A  N/A  N/A |

^a^Overall analyses encompassing time from screening through last WBCT scan; ^b^Spans the interruption due to the partial clinical hold and crossing the futility boundary, in patients who restarted palovarotene; ^c^Spans the time spent on treatment following interruptions with ≥2 WBCT scans post-restart; ^d^Spans the period from first WBCT scan off-treatment following dosing interruption, in patients who remained off treatment. max: maximum; min: minimum; N/A: not applicable; NHS: natural history study; SD: standard deviation.

## Table S5. Change from baseline in total CAJIS score in patients receiving palovarotene in MOVE and untreated patients from the NHS.

|  | **MOVE**  **(N=107)** | **NHS**  **(N=117)** |
| --- | --- | --- |
| Baseline^a^  n  Mean (SD)  Min, Max | 105  9.8 (5.93)  0.0, 25.0 | 100  11.9 (7.31)  1.0, 30.0 |
| Last visit  n  Mean (SD)  Min, Max | 105  11.5 (6.37)  0.0, 27.0 | 100  13.0 (7.39)  1.0, 30.0 |
| Change from baseline to last visit  n  Mean (SD)  Min, Max | 105  1.8 (3.06)  –4.0, 10.0 | 100  1.0 (2.66)  –7.0, 9.0 |
| Duration of of CAJIS assessments from baseline to last visit (months)  Mean (SD)  Median  Min, Max | 35.6 (14.980)  42.2  1.7, 51.4 | 26.3 (10.384)  25.4  10.0, 44.2 |

Includes patients who had a baseline and at least one post-baseline CAJIS score. ^a^Baseline was defined as screening visit in the NHS and latest visit on or prior to first treatment date in MOVE. CAJIS: cumulative analogue joint involvement scale; max: maximum; min: minimum; NHS: natural history study; SD: standard deviation.

## Table S6. Change from baseline in FOP-PFQ percentage of worst score in patients receiving palovarotene in MOVE and untreated patients from the NHS.

|  | **MOVE**  **(N=107)** | **NHS**  **(N=117)** |
| --- | --- | --- |
| Baseline^a^  n  Mean (SD)  Min, Max | 102  43.7 (26.1)  0.0, 98.2 | 101  46.6 (28.9)  0.0, 100.0 |
| Last visit  n  Mean (SD)  Min, Max | 102  50.5 (28.8)  0.0, 100.0 | 101  52.6 (28.7)  0.0, 100.0 |
| Change from baseline to last visit  n  Mean (SD)  Min, Max | 102  6.8 (14.9)  –28.6, 68.3 | 101  6.2 (11.9)  –23.1, 51.8 |
| Interval of FOP-PFQ Percentage of Worst Score from baseline to last visit (months)  Mean (SD)  Median  Min, Max | 30.4 (15.9)  35.2  1.6, 51.4 | 22.2 (12.8)  22.9  0.4, 44.2 |

Includes patients who had a baseline and at least one post-baseline FOP-PFQ score. ^a^Baseline was defined as first record per patient. FOP-PFQ: FOP-Physical Function Questionnaire; max: maximum; min: minimum; NHS: natural history study; SD: standard deviation.

## Table S7. Palovarotene exposure in patients receiving chronic and flare-up palovarotene treatment in MOVE.

| **Dosing parameter** | **Chronic treatment (N=99)** | **Flare-up treatment**  **(N=81)** | **Overall**  **(N=107)** |
| --- | --- | --- | --- |
| **Actual total dose, mg** |  |  |  |
| Mean (SD) | 2,329.5 (1,786.42) | 2,841.7 (2,613.84) | 4,480.7 (3,111.47) |
| Median (min, max) | 1,926.0 (27, 6,730) | 1,949.5 (163, 15,730) | 4,018.5 (133, 16,155) |
| **Duration of dosing, weeks^a^** |  |  |  |
| Mean (SD) | 77.0 (51.14) | 34.7 (26.68) | 103.3 (54.02) |
| Median (min, max) | 74.7 (1, 192) | 25.1 (2, 131) | 87.0 (4, 195) |
| **Duration of flare-up treatment, weeks**  High flare-up dosing  Mean (SD)  Median (min, max)  Low flare-up dosing  Mean (SD)  Median (min, max) | N/A  N/A  N/A  N/A | 17.1 (16.14)  11.7 (2, 98)  18.7 (13.40)  16.0 (0, 64) | 17.1 (16.14)  11.7 (2, 98)  18.7 (13.40)  16.0 (0, 64) |
| **Total exposure in MOVE, weeks^b^**  Mean (SD)  Median (min, max)  >30 months, n (%) | 170.2 (76.91)  183.3 (5, 264)  71 (66.4) | 178.1 (74.02)  209.3 (32, 264)  56 (69.1) | 170.2 (76.91)  183.3 (5, 264)  71 (66.4) |

^a^Duration of dosing (weeks): (last dose date - first dose date + 1)/7 – days without dosing. ^b^Total Exposure (weeks): (last date on study – first dose date + 1)/7. N/A: not applicable; SD: standard deviation.

## Table S8. TEAEs leading to permanent discontinuation of palovarotene in MOVE.

| **n (%)** | **Chronic treatment (N=99)** | **Flare-up treatment**  **(N=81)** | **Overall**  **(N=107)** |
| --- | --- | --- | --- |
| Any TEAE leading to permanent discontinuation of palovarotene | 8 (7.5) | 3 (3.7) | 11 (10.3) |
| Musculoskeletal and connective tissue disorders  Epiphyses premature fusion  Mobility decreased | 3 (2.8)  3 (2.8)  0 | 2 (2.5)  1 (1.2)  1 (1.2) | 5 (4.7)  4 (3.7)  1 (0.9) |
| Psychiatric disorders  Depression  Intentional self-injury | 2 (1.9)  1 (0.9)  1 (0.9) | 0  0  0 | 2 (1.9)  1 (0.9)  1 (0.9) |
| Skin and subcutaneous tissue disorders  Dry skin  Pruritus | 1 (0.9)  0  1 (0.9) | 1 (1.2)  1 (1.2)  0 | 2 (1.9)  1 (0.9)  1 (0.9) |
| Infections and infestations  Furuncle | 1 (0.9)  1 (0.9) | 0  0 | 1 (0.9)  1 (0.9) |
| Investigations  Amylase increased  Lipase increased | 1 (0.9)  1 (0.9)  1 (0.9) | 0  0  0 | 1 (0.9)  1 (0.9)  1 (0.9) |
| Metabolism and nutrition disorders  Malnutrition | 0  0 | 1 (1.2)  1 (1.2) | 1 (0.9)  1 (0.9) |

Data are presented as number (%) of patients. Treatment-emergent was defined as any AE with an onset date on or after first dose date of chronic treatment and up to seven days past the date of last dose of chronic treatment. AE: adverse event; TEAE: treatment-emergent AE.

## Table S9. Concomitant medications taken within 3 days of skin TEAE onset.

| **Anatomic Therapeutic Class Level 3**  **Preferred Term, n (%)** | **Overall**  **(N=97)** |
| --- | --- |
| Any concomitant medication taken within 3 days of skin TEAE onset | 89 (91.8) |
| **Emollients and protectives**  Other emollients and protectives  Soft paraffin and fat products  Dimeticone | **65 (67.0)**  36 (37.1)  26 (26.8)  10 (10.3) |
| **Corticosteroids, plain**  Hydrocortisone  Triamcinolone  Aloe vera with Avena sativa/hydrocortisone/Vitam | **42 (43.3)**  24 (24.7)  10 (10.3)  5 (5.2) |
| **Corticosteroids for systemic use, plain**  Prednisone  Prednisolone  Methylprednisolone | **34 (35.1)**  25 (25.8)  5 (5.2)  4 (4.1) |
| **Antihistamines for systemic use**  Desloratadine  Hydroxyzine  Cetirizine | **32 (33.0)**  9 (9.3)  7 (7.2)  6 (6.2) |
| **Anti-inflammatory and anti-rheumatic products, non-steroids**  Ibuprofen  Naproxen | **21 (21.6)**  17 (17.5)  3 (3.1) |

Data are presented as number of patients (percentage of patients). The five most common Anatomic Therapeutic Classes, and within those, the three most common Preferred Terms used by >1 patient are reported. If a patient experienced more than one event in a category, the patient was counted only once in that category. TEAE: treatment-emergent adverse event.

## Table S10. Concomitant medications taken within 3 days of onset for the most common skin TEAEs.

|  | Chronic dosing, with prophylaxis | Chronic dosing, without prophylaxis | Flare-up dosing, with prophylaxis | Flare-up dosing, without prophylaxis |
| --- | --- | --- | --- | --- |
| Dry skin  Number of events  Concomitant medications, n (%)  Prednisone  Other emollients and protectives | 54  2 (3.7)  4 (7.4) | 37  1 (2.7)  9 (24.3) | 37  6 (16.2)  1 (2.7) | 39  14 (35.9)  7 (17.9) |
| Dry lips  Number of events  Concomitant medications, n (%)  Other emollients and protectives  Prednisone  Methylprednisolone  Avena sativa fluid extract | 15  4 (26.7)  0  0  0 | 20  7 (35.0)  0  0  1 (5.0) | 12  0  4 (33.3)  1 (8.3)  0 | 5  1 (20.0)  1 (20.0)  1 (20.0)  1 (20.0) |
| Alopecia  Number of events  Concomitant medications, n (%)  Prednisone | 8  0 | 13  0 | 13  3 (23.1) | 12  2 (16.7) |
| Drug eruption  Number of events  Concomitant medications, n (%)  Hydrocortisone  Other emollients and protectives  Desloratadine | 13  3 (23.1)  1 (7.7)  0 | 10  1 (10.0)  3 (30.0)  3 (30.0) | 26  1 (3.8)  3 (11.5)  0 | 14  0  1 (7.1)  3 (21.4) |
| Pruritus  Number of events  Concomitant medications, n (%)  Other emollients and protectives  Loratadine  Chlorphenamine maleate  Calamine/01864001  Emla/00675501  Hydrocortisone acetate | 21  6 (28.6)  0  0  0  0  0 | 4  1 (25.0)  0  1 (25.0)  1 (25.0)  1 (25.0)  1 (25.0) | 8  0  2 (25.0)  1 (12.5)  0  0  0 | 6  1 (16.7)  0  0  0  0  0 |
| Rash  Number of events  Concomitant medications, n (%)  Hydrocortisone  Triamciclone  Chlorphenamine maleate  Hydrocortisone acetate  Calamine/01864001  Ibuprofen | 18  4 (22.2)  5 (27.8)  0  1 (5.6)  0  0 | 15  0  0  3 (20.0)  5 (33.3)  3 (20.0)  0 | 13  1 (7.7)  1 (7.7)  1 (7.7)  0  0  0 | 6  3 (50.0)  0  0  0  0  2 (33.3) |
| Erythema  Number of events  Concomitant medications, n (%)  Other emollients and protectives  Aloe vera  Soft paraffin and fat products  Desloratadine  Boric acid  Hydroxyzine  Prednisone | 9  5 (55.6)  3 (33.3)  2 (22.2)  0  0  0  0 | 6  0  0  0  1 (16.7)  0  0  0 | 15  0  0  0  1 (6.7)  0  0  0 | 5  0  0  0  1 (20.0)  1 (20.0)  1 (20.0)  1 (20.0) |
| Pruritus generalized  Number of events  Concomitant medications, n (%)  Heparinoid  Hydrocortisone  Ebastine | 9  0  3 (33.3)  0 | 11  4 (36.4)  3 (27.3)  0 | 14  0  0  0 | 4  0  0  1 (25.0) |
| Skin exfoliation  Number of events  Concomitant medications, n (%)  Soft paraffin and fat products  Other emollients and protectives  Nivea/ 01338601  Pyrithione zinc | 10  2 (20.0)  1 (10.0)  0  0 | 3  0  1 (33.3)  1 (33.3)  1 (33.3) | 20  2 (10.0)  0  0  0 | 2  0  0  0  0 |
| Skin irritation  Number of events  Concomitant medications, n (%)  Soft paraffin and fat products  Bacitracin/neomycin/polymy nxin B/ zinc  Ciprofloxacin hydrochloride  Keluamid  Lipikar/02826001  Mometasone furoate  Prednisone  Topical protectives against UV radiation | 8  1 (12.5)  0  0  0  0  0  0  0 | 1  0  0  0  1 (100.0)  1 (100.0)  0  0  1 (100.0) | 2  1 (50.0)  1 (50.0)  0  0  0  0  0  0 | 3  1 (33.3)  0  1 (33.3)  0  0  1 (33.3)  1 (33.3)  0 |

Data for concomitant medications used for ≥20% of skin TEAEs are reported. One TEAE could only occur during chronic or flare-up treatment. TEAE: treatment-emergent adverse event.

## Table S11. Dosing adjustments made to manage the most common skin TEAEs in patients receiving chronic and flare-up palovarotene treatment, with and without prophylaxis.

| **Action taken by Preferred Term, n/N (%)** | **Chronic treatment** | | **Flare-up treatment** | |
| --- | --- | --- | --- | --- |
|  | With prophylaxis | Without prophylaxis | With prophylaxis | Without prophylaxis |
| Dry skin  Dose not changed  Dose modified  Drug interrupted  Drug withdrawn | 53/54 (98.1)  1/54 (1.9)  0  0 | 36/37 (97.3)  1/37 (2.7)  0  0 | 34/37 (91.9)  2/37 (5.4)  0  1/37 (2.7) | 27/39 (69.2)  12/39 (30.8)  0  0 |
| Dry lips  Dose not changed  Dose modified  Drug interrupted  Drug withdrawn | 14/15 (93.3)  1/15 (6.7)  0  0 | 19/20 (95.0)  1/20 (5.0)  0  0 | 11/12 (91.7)  1/12 (8.3)  0  0 | 5/5 (100.0)  0  0  0 |
| Alopecia  Dose not changed  Dose modified  Drug interrupted  Drug withdrawn | 8/8 (100.0)  0  0  0 | 13/13 (100.0)  0  0  0 | 11/13 (84.6)  2/13 (15.4)  0  0 | 12/12 (100.0)  0  0  0 |
| Drug eruption  Dose not changed  Dose modified  Drug interrupted  Drug withdrawn | 9/13 (69.2)  2/13 (15.4)  2/13 (15.4)  0 | 8/10 (80.0)  2/10 (20.0)  0  0 | 16/26 (61.5)  9/26 (34.6)  1/26 (3.8)  0 | 7/14 (50.0)  6/14 (42.9)  1/14 (7.1)  0 |
| Pruritus  Dose not changed  Dose modified  Drug interrupted  Drug withdrawn | 19/21 (90.5)  0  1/21 (4.8)  1/21 (4.8) | 3/4 (75.0)  0  1/4 (25.0)  0 | 7/8 (87.5)  1/8 (12.5)  0  0 | 3/6 (50.0)  3/6 (50.0)  0  0 |
| Rash  Dose not changed  Dose modified  Drug interrupted  Drug withdrawn | 17/18 (94.4)  0  1/18 (5.6)  0 | 12/15 (80.0)  0  3/15 (20.0)  0 | 10/13 (76.9)  1/13 (7.7)  2/13 (15.4)  0 | 6/6 (100.0)  0  0  0 |
| Erythema  Dose not changed  Dose modified  Drug interrupted  Drug withdrawn | 9/9 (100.0)  0  0  0 | 6/6 (100.0)  0  0  0 | 12/15 (80.0)  3/15 (20.0)  0  0 | 5/5 (100.0)  0  0  0 |
| Pruritus generalized  Dose not changed  Dose modified  Drug interrupted  Drug withdrawn | 9/9 (100.0)  0  0  0 | 11/11 (100.0)  0  0  0 | 8/14 (57.1)  5/14 (35.7)  1/14 (7.1)  0 | 3/4 (75.0)  1/4 (25.0)  0  0 |
| Skin exfoliation  Dose not changed  Dose modified  Drug interrupted  Drug withdrawn | 9/10 (90.0)  1/10 (10.0)  0  0 | 3/3 (100.0)  0  0  0 | 15/20 (75.0)  5/20 (25.0)  0  0 | 2/2 (100.0)  0  0  0 |
| Skin irritation  Dose not changed  Dose modified  Drug interrupted  Drug withdrawn | 7/8 (87.5)  1/8 (12.5)  0  0 | 1/1 (100.0)  0  0  0 | 2/2 (100.0)  0  0  0 | 3/3 (100.0)  0  0  0 |

Data are presented as number of patients/number of events (percentage of patients); percentages are expressed with the number of events as the denominators, respectively. TEAE: treatment-emergent adverse event.

## Table S12. Baseline demographics and characteristics of patients receiving palovarotene in MOVE and the phase II OLE, and untreated patients from the NHS, included in biomechanical computed tomography analyses.

|  | **NHS (N=114)** | **MOVE**  **(N=107)** | **Phase II OLE (N=54)** |
| --- | --- | --- | --- |
| **Demographics** | | | |
| Sex, female, n (%) | 52 (45.6) | 49 (45.8) | 31 (57.4) |
| Age, mean ± SD | 17.6 ± 9.7 | 15.5 ± 10.1 | 21.0 ± 9.2 |
| Age, ≥18 years, n (%) | 48 (42.1) | 27 (25.2) | 34 (63.0) |
| Age at FOP onset, years, mean ± SD | 6.5 ± 5.1 | 5.9 ± 4.8 | 5.2 ± 4.7 |
| **Treatment characteristics** | | | |
| Steroid use, n (%) | 85 (75.89) | 64 (59.81) | 33 (61.11) |
| **Bone Characteristics** | | | |
| Baseline CAJIS, mean ± SD | 11.7 ± 7.3 | 9.8 ± 5.9 | 11.7 ± 3.5 |
| Baseline HO (×10^3^ mm^3^), mean ± SD | 15.8 ± 14.2 | 15.0 ± 18.9 | 18.2 ± 16.1 |
| Vertebral body strength (newtons), mean ± SD | 4,512.2 ± 1,561.8 | 4,316.8 ± 1,507.3 | 4,699.1 ± 2,027.8 |
| Entire vertebral body bone mineral content (g), mean ± SD | 4.4 ± 2.0 | 4.1 ± 1.7 | 5.1 ± 2.1 |
| Mid-vertebral (trabecular) density (mg/cm^3^), mean ± SD | 103.8 ± 35.8 | 105.9 ± 38.3 | 93.2 ± 33.0 |
| **Vertebral Fractures** | | | |
| Any fracture at baseline, n (%) | 25 (23.6) | 20 (21.5) | 10 (21.3) |
| Number of fractures at baseline, n (%)^a^  0  1  2  3  4  5+ | 81 (76.4)  13 (12.3)  7 (6.6)  3 (2.8)  1 (0.9)  1 (0.9) | 73 (78.5)  16 (17.2)  2 (2.2)  1 (1.1)  0 (0.0)  1 (1.1) | 37 (78.7)  8 (17.0)  1 (2.1)  1 (2.1)  0 (0.0)  0 (0.0) |
| Any moderate/severe fractures at baseline, n (%) | 13 (12.3) | 7 (7.5) | 1 (2.1) |
| Number of moderate/severe fractures at baseline, n (%)^a^  0  1  2  3  4  5+ | 93 (87.7)  9 (8.5)  3 (2.8)  1 (0.9)  0 (0.0) 0 (0.0) | 86 (92.5)  7 (7.5)  0 (0.0)  0 (0.0)  0 (0.0)  0 (0.0) | 46 (97.9)  1 (2.1)  0 (0.0)  0 (0.0)  0 (0.0)  0 (0.0) |

^a^Data were available for 106 (93.00%) patients in the NHS, 93 (86.90%) patients in MOVE and 47 (87.00%) patients in the phase II OLE. CAJIS: cumulative analogue joint involvement scale; FOP: fibrodysplasia ossificans progressiva; HO: heterotopic ossification; NHS: natural history study; OLE: open-label extension; SD: standard deviation.

## Table S13. Adjusted regression model calculated change in vertebral body strength, entire vertebral body BMC and mid-vertebral (trabecular) density per year by effect in patients receiving palovarotene in the phase II OLE (N=45) versus untreated patients from the NHS (N=85).

|  | **Mid-vertebral (trabecular) density,** **mg/cm^3^** | **Entire vertebral body BMC, g** | **Vertebral body strength, newtons** |
| --- | --- | --- | --- |
| Sex, female | 1.05  (–0.78, 2.88) | 0.00  (–0.08, 0.07) | 23.98  (–103.99, 151.95) |
| Age at FOP symptom onset | 0.18^a^  (0.01, 0.35) | 0.00  (–0.01, 0.01) | –1.14  (–15.70, 13.43) |
| Steroid use | 0.14  (–1.84, 2.12) | 0.03  (–0.04, 0.11) | –8.86  (–111.11, 93.38) |
| Baseline CAJIS score | 0.10  (–0.13, 0.33) | –0.01^b^  (–0.02, –0.01) | –11.05  (–34.71, 12.62) |
| Palovarotene treatment | –0.04  (–1.87, 1.78) | –0.15^b^  (–0.23, –0.06) | –180.18  (–365.25, 4.89) |

Data are presented as beta (95% CI). ^a^p<0.05; ^b^p<0.001. BMC: bone mineral content; CAJIS: cumulative analogue joint involvement scale; FOP: fibrodysplasia ossificans progressiva; OLE: open-label extension.

# References

1.	Pignolo RJ, Al Mukaddam M, Baujat G, et al. Study methodology and insights from the palovarotene clinical development program in fibrodysplasia ossificans progressiva. BMC Medical Research Methodology 2023;23:269.

2.	Pignolo RJ, Hsiao EC, Al Mukaddam M, et al. Reduction of New Heterotopic Ossification (HO) in the Open-Label, Phase 3 MOVE Trial of Palovarotene for Fibrodysplasia Ossificans Progressiva (FOP). J Bone Miner Res 2023;38:381-394.
